# Burden of oral cancer in Asia from 1990 to 2019: Estimates from the Global Burden of Disease 2019 study

**DOI:** 10.1371/journal.pone.0265950

**Published:** 2022-03-24

**Authors:** Long Xie, Zhengjun Shang

**Affiliations:** 1 The State Key Laboratory Breeding Base of Basic Science of Stomatology (Hubei-MOST) & Key Laboratory of Oral Biomedicine Ministry of Education, School & Hospital of Stomatology, Wuhan University, Wuhan, China; 2 Department of Oral and Maxillofacial-Head and Neck Oncology, School and Hospital of Stomatology, Wuhan University, Wuhan, China; University of New South Wales, AUSTRALIA

## Abstract

**Background:**

Oral cancer (OC) poses a threat to human health and imposes a heavy burden on countries. We assessed the burden imposed by OC on Asian nations from 1990 to 2019 based on gender and age.

**Methods:**

We collected oral cancer data from the 2019 Global Burden of Disease study from 1990 to 2019 in 45 Asian countries and territories. Annual case data and age-standardised rates (ASRs) were used to investigate the incidence, mortality, and disability-adjusted life-years (DALYs) of OC based on age and gender from 1990 to 2019 in 45 Asian countries and territories. Estimated annual percentage changes (EAPCs) were used to assess incidence rate, mortality, and trends in DALYs.

**Results:**

The age-standardised incidence rate (ASIR) of OC increased from 1990 to 2019 with an EAPC of 0.32 (95% CI, 0.19–0.46), and the age-standardised death rate of OC remained stable at an EAPC of 0.08 (95%CI, from -0.06 to 0.21). The age-standardised DALYs of OC decreased at an EAPC of -0.16 (95%CI, from -0.30 to -0.02). The proportion of patients older than 70 years increased yearly in terms of incidence, mortality, and DALYs from 1990 to 2019. Of the DALYs, smoking was the main contributor in the Asian regions, and the largest contributor to DALYs in most Asian regions. Other contributors were alcohol use and chewing tobacco.

**Conclusion:**

Although the burden of OC was declining in Asia, South Asia remained the region with the highest burden. OC caused the greatest burden in Pakistan, Taiwan China, and India. Therefore, measures should be taken to reduce the burden of oral cancer in high-risk regions and countries with attributable risk factors.

## Introduction

Oral cancer (OC) is a malignant lesion occurring in the oral cavity and the sixth most common cancer in the world [1]. More than 389,760 new cases and 193,696 death cases of OC were recorded in 2017 worldwide, and the incidence and mortality of OC had increased yearly from 1990 to 2017 [2]. Although different treatments are available, the overall five-year survival rate of OC (all stages included) is only approximately 50% [3]. The survival of patients with OC and recurrence of the disease is closely related to lymph node metastasis and the spread of tumours [4]. Risk factors associated with OC include smoking, alcohol, ultraviolet light, human papillomavirus, betel quid use, insufficient intake of fruits and vegetables, periodontal disease, and cooking oil fumes [5–7].

Access to cancer services, financial constraints, social and cultural factors, and lack of early diagnosis and screening services contribute to global differences in cancer outcomes [8]. Asia, especially South Asia, had the highest incidence, mortality and national burden of OC in 2017 [2]. The annual cost of treating OC from its pre-cancerous stage to its advanced stage is increasing [9]. The economic cost of treating oral cancer is huge, placing a heavy burden on families and the health care system [10]. Governments may fail in formulating public health policies to mitigate this disease because of insufficient data on the trends in incidence, mortality, and disability-adjusted life years (DALYs). As far as we know, no comprehensive report has examined the incidence, death, and secular trends of OC in Asia.

The Global Burden of Disease Study 2019 (GBD 2019) is a comprehensive study of health loss designed to capture complex patterns of disease and injury burden. It has helped policymakers in Asian countries assess the burden of OC correctly and allocate the finite resources of health care systems [11]. Therefore, this study aimed to assess the burden imposed by OC on Asian nations from 1990 to 2019 based on gender and age. This study may help to provide information support for Asian governments to formulate reasonable OC-related health policies.

## Materials and methods

### Study data

Data on the burden imposed by OC on Asian countries (44 countries and one territory included) were obtained from the online data source Global Health Data Exchange (GHDx) query tool (http://ghdx.healthdata.org/gbd-results-tool), which is the product of ongoing global collaboration and uses all available epidemiological data for the comparative assessment of health loss due to 364 diseases across 204 countries and territories. Furthermore, we used gender and age information to assess their effect on the burden of OC. It should be noted that individuals that were younger than five years were excluded because of insufficient data. The GBD 2019 provides data on the annual morbidity and mortality, DALYs, and respective age-standardised rate (ASR) of oral cancer from 1990 to 2019. In particular, we use the data of North Africa and the Middle East as a proxy for West Asia, since 17 of the 21 countries in the GBD database for North Africa and the Middle East belong to West Asia.

### Statistical analysis

The annual age-standardised incidence rate (ASIR), age-standardised death rate (ASDR), age-standardised DALYs, and estimated annual percentage changes (EAPCs) were calculated and used in assessing the trends in the incidence and mortality of OC. DALYs were calculated by obtaining the sum of the years lived with disability and the years of life lost [12]. ASR was obtained using the GHDx query tool and used as an objective indicator for quantifying the trend in cancer incidence. EAPC was used to describe the trend in ASR within a specified time interval [13]. The natural logarithm of ASR has a linear relationship with time, i.e., Y = α+βX+ε, where Y refers to ln (ASR), X stands for the calendar year, and ε represents error/noise term. In the formula, β is the slope, indicating a linear-positive or negative trend in ASR. EAPC was calculated using the formula EAPC = 100*(exp (β)-1); The formula of EAPC and its 95% confidence interval were obtained using a linear model. When the lower limit of the confidence interval and the EAPC were positive, ASR was considered to have an upward trend and vice versa. All statistical analyses were performed using R software (version 4.0.2). A *p* value of < 0.05 was considered statistically significant.

## Results

### Analysis of OC incidence in Asia

**Fig 1:**
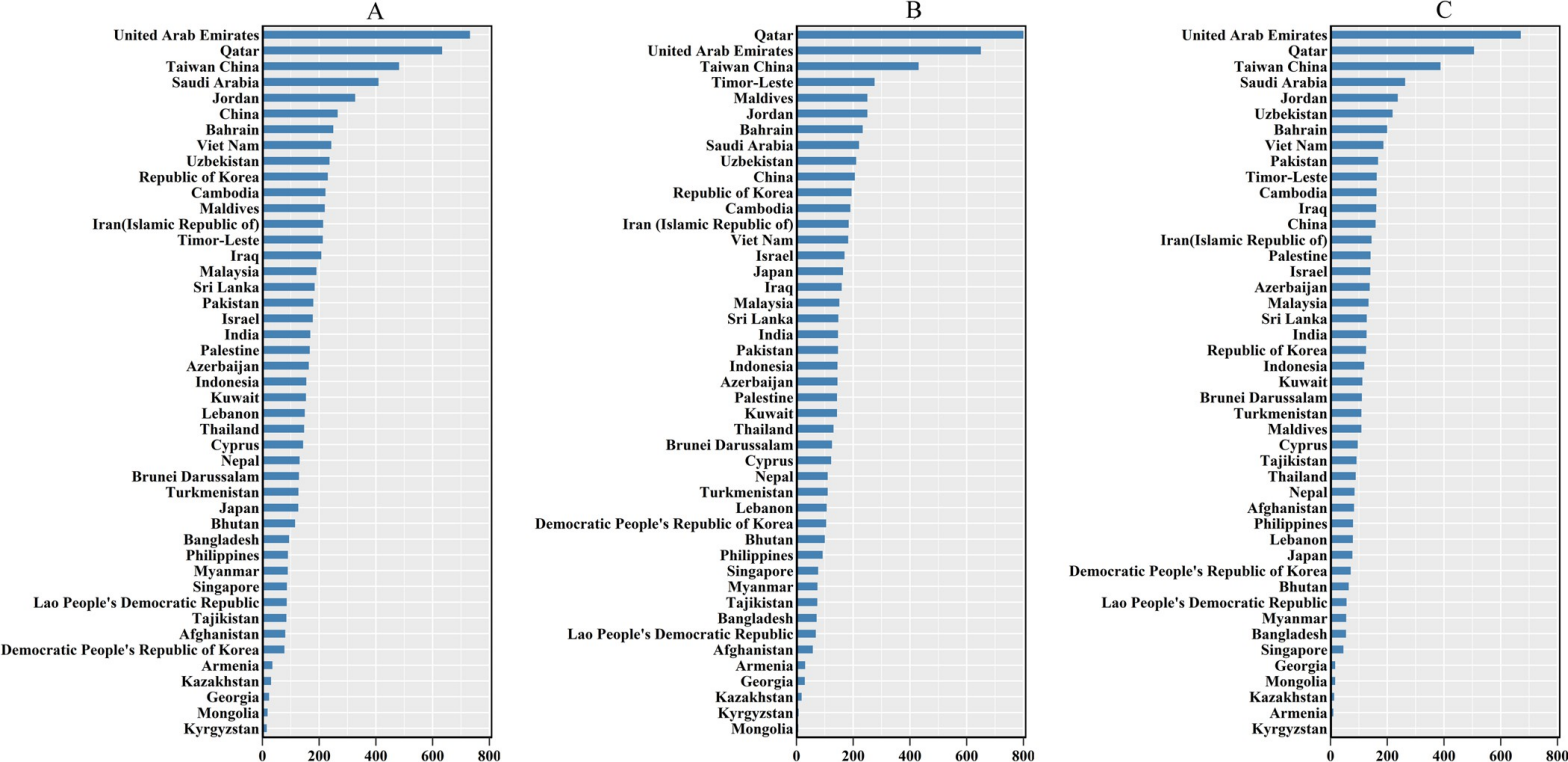
The relative change in burden of oral cancer in Asia. (a) The relative change in incidences of oral cancer between 1990 and 2019; (b) The relative change in deaths of oral cancer between 1990 and 2019; (c) The relative change in DALYs of oral cancer between 1990 and 2019.

**Fig 2:**
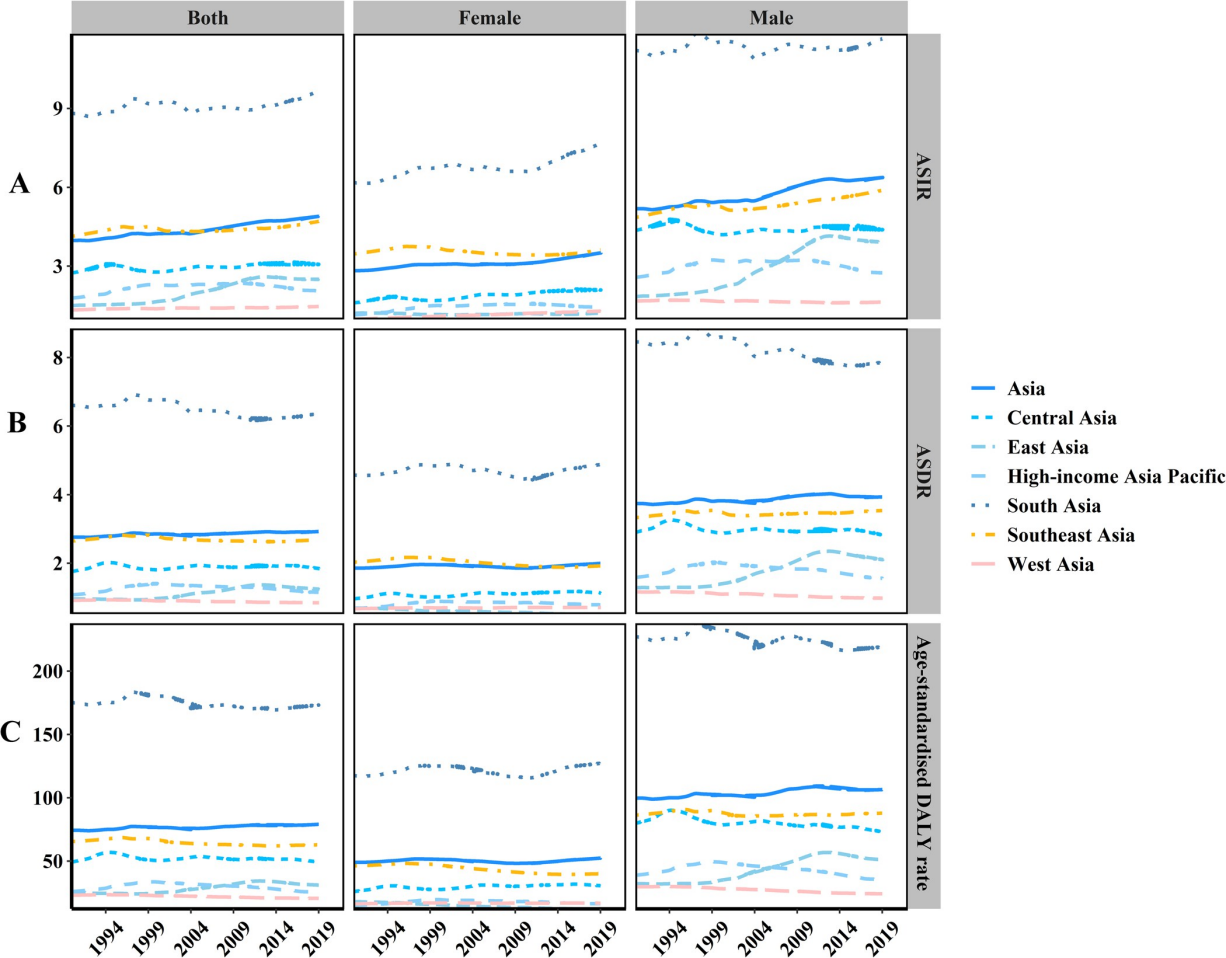
The change trends of age-standardised incidence, death, and DALY rate in Asia regions between 1990 and 2019. (A) ASIR, (B) ASDR, (C) Age-standardised DALY rate.

**Fig 3:**
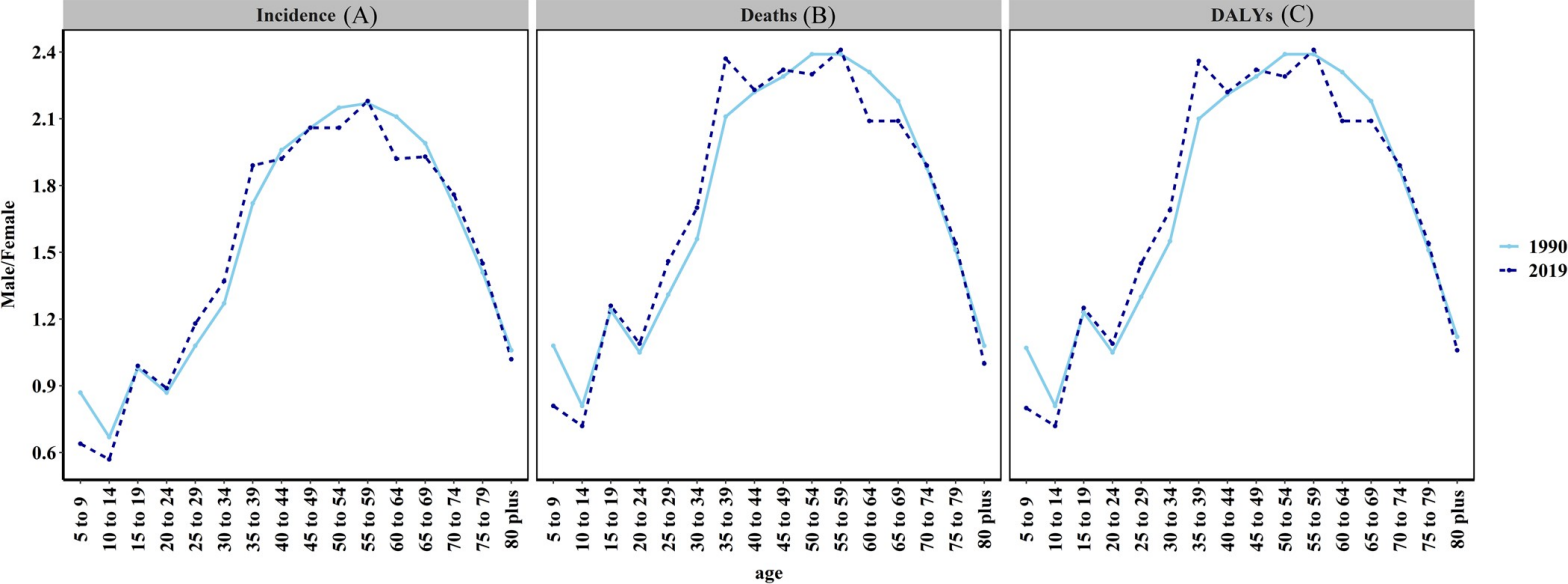
The ratio of male to female incidence, deaths, and DALYs among different age groups in 1990 and 2019. (a) The ratio of male to female incidence; (b) The ratio of male to female deaths (c) The ratio of male to female DALYs.

The incident cases of OC increased from 86,413 in 1990 to 239,759 in 2019, with a total increase of 177.46%. The ASIR displayed an upward trend at an EAPC of 0.32 (95% CI, 0.19–0.46), increasing from 3.98 to 4.90 per 100,000 persons (S1 Table). The number of OC cases on average was 152,542 (129,747–176,233) in males, which was 0.75-fold higher than that in females 87,217 (ranging from 76,467 to 98,928). The increased time in the OC incidence cases from 1990 to 2019 varied by country (Fig 1A, S1 Table). Over the past 30 years, ASIR in males was nearly twice that in females (Fig 2A). The male-female incidence ratio manifested a bimodal distribution in different age groups in 1990 and 2019, and the peaks were observed in the 15–19- and 55–59-year age groups (Fig 3A).

In terms of geographic regions, the highest ASIR was found in South Asia (9.65/100,000, 95%CI = 8.17–11.15/100,000) in 2019 and the lowest in West Asia (1.46/100,000, 95%CI = 1.28–1.68/100,000). Furthermore, ASIR was on the rise in all Asian regions. (Fig 2A, S1 Table)

**Fig 4:**
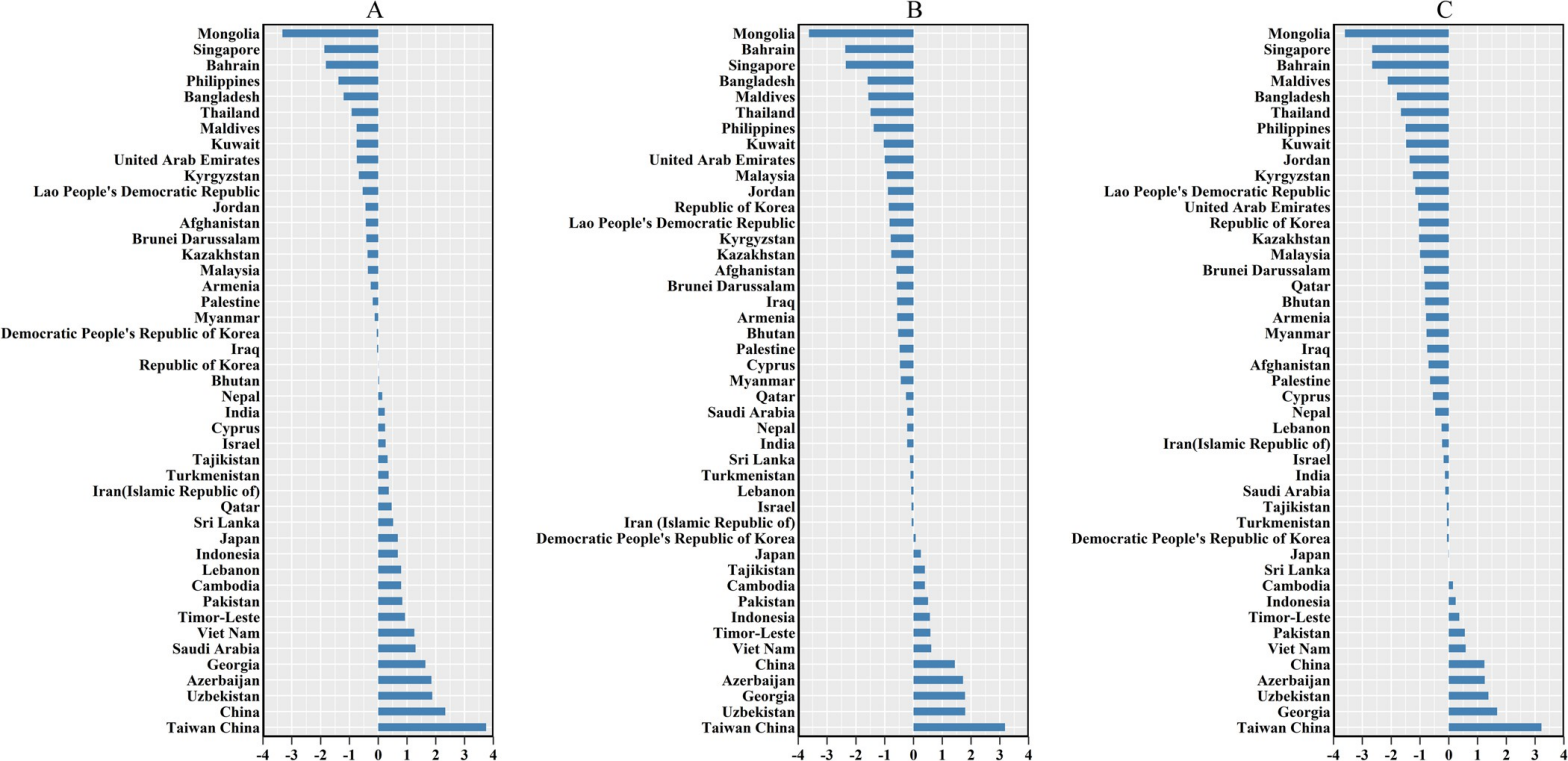
The EAPC of oral cancer ASR from 1990 to 2019. (a) The EAPC of oral cancer ASIR from 1990 to 2019; (b) The EAPC of oral cancer ASDR from 1990 to 2019; (c) The EAPC of oral cancer age-standardised DALY from 1990 to 2019.

The three countries with the highest incidence cases of OC in 2019 were India (104,838), China (45,216), and Pakistan (28,579), and the countries with the lowest cases were Brunei Darussalam (16), Maldives (16), and Bahrain (21) as shown in S2 Table. The three countries or territories with the highest ASIRs were Pakistan (21.93 per 100,000), Taiwan China (16.35/100,000), and India (8.82/100,000), and the countries with the lowest ASIR were Iran (1.26/100,000), Palestine (1.28/100,000) and Kuwait (1.36/100,000). The number of OC incidence cases increased in all Asian countries or territories, but the EAPC of ASIR in 17 countries was less than zero, and the EAPC of 21 countries or territories was greater than 0, while the trend of ASIR in seven countries remained stable (S1 Table). It can be seen from Fig 4A that the ASIR of OC in Taiwan China has the fastest growth rate.

**Fig 5:**
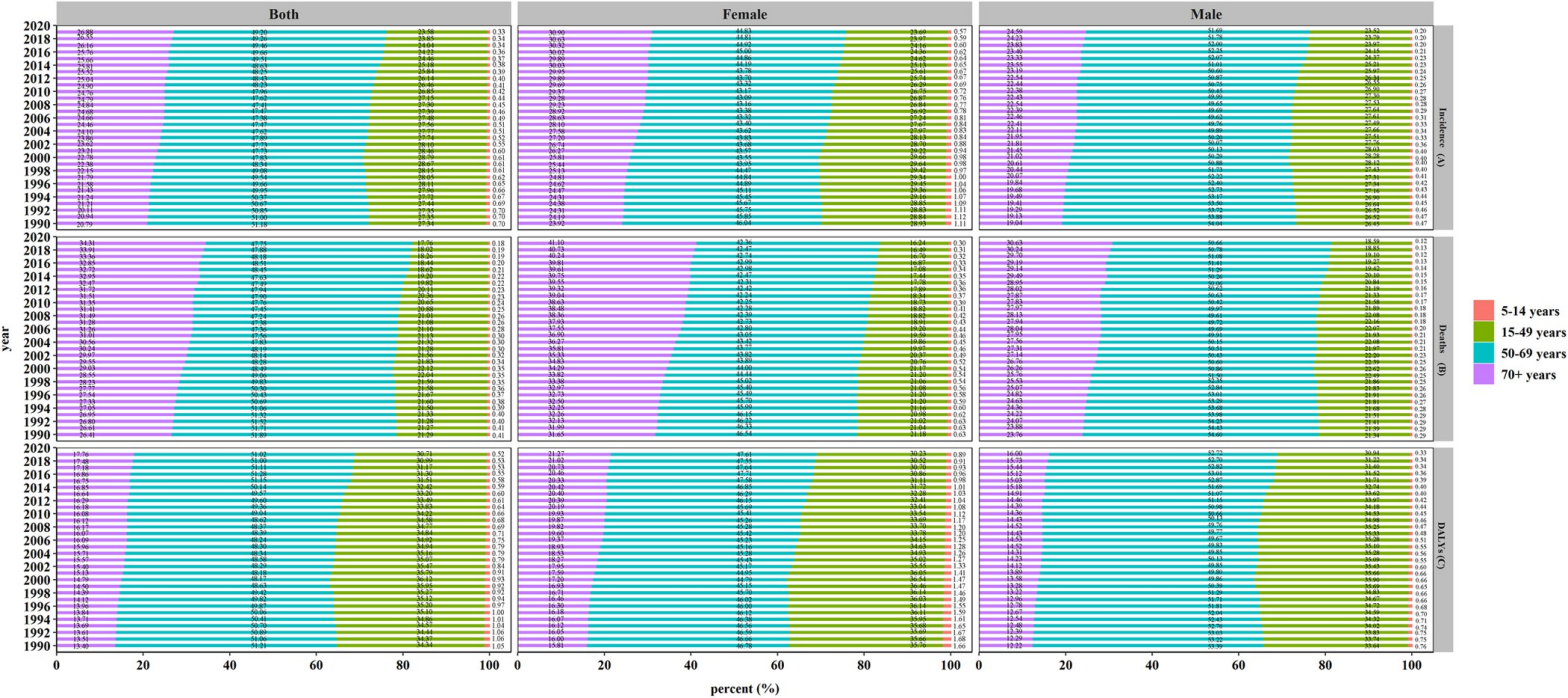
The proportion of different age groups in oral cancer between 1990 and 2019. (A) incidence, (B) death, (C) DALYs.

Young cases constituted a small proportion of OC cases, but the proportion of women older than 70 years increased by 6.98% from 1990 to 2019 (Fig 5A).

### Analysis of OC death in Asia

The number of OC deaths in Asia increased by 148.76%, but the ASDR of OC showed a stable trend at an EAPC of 0.08 (95%CI, from -0.06 to 0.21), from 2.76/100,000 persons in 1990 to 2.93/100,000 persons in 2019 (Fig 2B, S3 Table). The three countries and territories with the highest increase in OC deaths from 1990 to 2019 were Qatar, the United Arab Emirates (UAE), and Taiwan China (Fig 1B, S3 Table). ASDR in males was twice that in females (Fig 2B, S3 Table). The male-female incidence ratio indicated a bimodal distribution in different age groups in 1990 and a trimodal distribution in 2019 (Fig 3B).

In terms of geographic regions, the highest ASDR was in South Asia (6.36/100,000, 95%CI = 5.48–7.39/100,000) in 2019 and the lowest in West Asia (0.85/100,000, 95%CI = 0.75–0.97/100,000). Of all the regions in Asia, only the ASDR in East Asia showed an upward trend with an EAPC of 1.53 (95%CI = 1.27–1.80) as shown in S3 Table.

In 2019, Pakistan, Taiwan China, and India had the highest ASDRs, whereas India, China, and Pakistan had the highest number of death cases (S2 Table). Although the number of OC death cases increased in all Asian countries, Mongolia, Kyrgyzstan, and Kazakhstan had the lowest increases (Fig 1B). Furthermore, cases in females in Mongolia decreased by 27.27%, and cases in males in Kyrgyzstan decreased by 1.96% from 1990 to 2019 (S3 Table). The highest EAPCs of ASDR in both genders were obtained in Taiwan China, and the lowest in Mongolia (Fig 4B, S2 and S3 Tables).

The proportion of deaths among people over 70 years of age increased from 26.41% in 1990 to 34.31% in 2019 (Fig 5B).

### Analysis of OC DALYs in Asia

The DALYs of OC increased from 1,723,076 (95% UI = 1,568,080–1,901,321) in 1990 to 3,954,826 (95% UI = 3,500,348–4,470,867), and the age-standardised DALYs showed a slight downward trend at an EAPC of -0.16 (95%CI, from—0.30 to—0.02) as shown in Fig 2C and S4 Table. The three countries and territories with the highest increase in the number of OC DALYs from 1990 to 2019 were UAE, Qatar, and Taiwan China. (Fig 1C, S2 and S4 Tables), and the trends in age-standardised DALYs of OC and those in the distribution of male-to-female ratio in different age groups were the same as the trend in ASDR (Figs 2C, 3B and 3C).

In terms of geographic regions, the highest age-standardised DALY was in South Asia (173.17/100,000, 95% CI = 149.26–202.43/100,000) in 2019 and the lowest in West Asia (20.62/100,000, 95%CI = 17.95–23.91/100,000). Of all the regions in Asia, only the age-standardised DALY in East Asia showed an upward trend with an EAPC 1.37 (95%CI = 1.11–1.62) as shown in S4 Table.

In 2019, the highest age-standardised DALYs were found in Pakistan (421.87/100,000), and the lowest was found in the Kuwait (15.30/100,000) (S2 Table). The countries with the smallest increases in the total number of DALYs were Kyrgyzstan (3.16%), Armenia (9.68%), and Kazakhstan (12.88%) (Fig 1C, S4 Table), but the number of DALYs declined among females in Mongolia (−21.18%), Georgia (−8.27%) and Armenia (−5.17%) and males in Kyrgyzstan (−6.52%) (S4 Table). The highest EAPCs in the age-standardised DALYs for both genders were found in Taiwan China, and the lowest in Mongolia (Fig 4C, S2 Table).

The proportion of OC DALYs among people over 70 years old increased from 13.40% in 1990 to 17.76% in 2019. Females older than 70 had the largest increase (5.47%) (Fig 5C).

### OC burden in Asia attributable to risk factors

Table 1 shows the percentage various risk factors contribute to the burden of oral cancer in Asia, such as chewing tobacco, smoking, and alcohol use.

**Table 1:** Risk factors attributable to OC burden in Asia in 1990 and 2019.

Location Risk		Asia	Central Asia	East Asia	High-income Asia Pacific	West Asia	South Asia	Southeast Asia
Chewing tobacco, percent (95% CI)	1990	24.99(19.00,31.01)	2.65(1.58,4.05)	1.21(0.75,1.83)	1.52(0.97,2.21)	4.24(2.70,6.04)	34.28(26.04,42.63)	11.30(8.56,14.43)
	2019	25.08(19.36,30.78)	3.54(2.03,5.40)	1.27(0.73,2.02)	1.70(1.10,2.46)	4.24(2.70,6.04)	34.72(27.01,42.54)	11.11(8.39,14.17)
Smoking, percent (95% CI)	1990	30.72(24.03,36.8)	37.78(31.89,43.04)	33.57(27.48,39.09)	47.66(41.62,53.26)	32.16(27.05,37.16)	28.50(21.58,35.41)	33.98(26.13,40.76)
	2019	27.65(21.62,32.9)	33.02(27.35,38.15)	45.37(39.05,51.5)	36.49(31.08,42.00)	28.94(24.05,33.30)	21.94(15.83,27.33)	34.28(26.73,41.03)
Alcohol use, percent (95% CI)	1990	19.10(14.85,23.76)	35.14(28.03,42.18)	32.58(26.26,38.53)	47.53(38.99,54.59)	8.94(6.61,11.45)	14.50(10.38,19.26)	17.42(13.32,21.44)
	2019	25.66(19.95,30.84)	34.54(27.88,40.93)	43.30(35.75,50.47)	42.03(33.58,50.03)	7.66(5.51,10.02)	20.12(14.55,25.79)	29.84(24.37,34.96)
All risk factors, percent (95% CI)	1990	57.91(52.53,62.81)	57.42(52.3,62.47)	51.40(45.86,56.79)	71.64(66.34,76.08)	39.61(34.97,44.29)	59.72(53.46,65.19)	51.31(45.07,56.96)
	2019	60.20(55.11,65.11)	54.32(49.22,59.03)	66.36(60.99,71.39)	62.20(56.09,67.71)	35.87(31.66,39.91)	59.33(53.19,64.99)	58.86(53.44,63.85)

**Fig 6:**
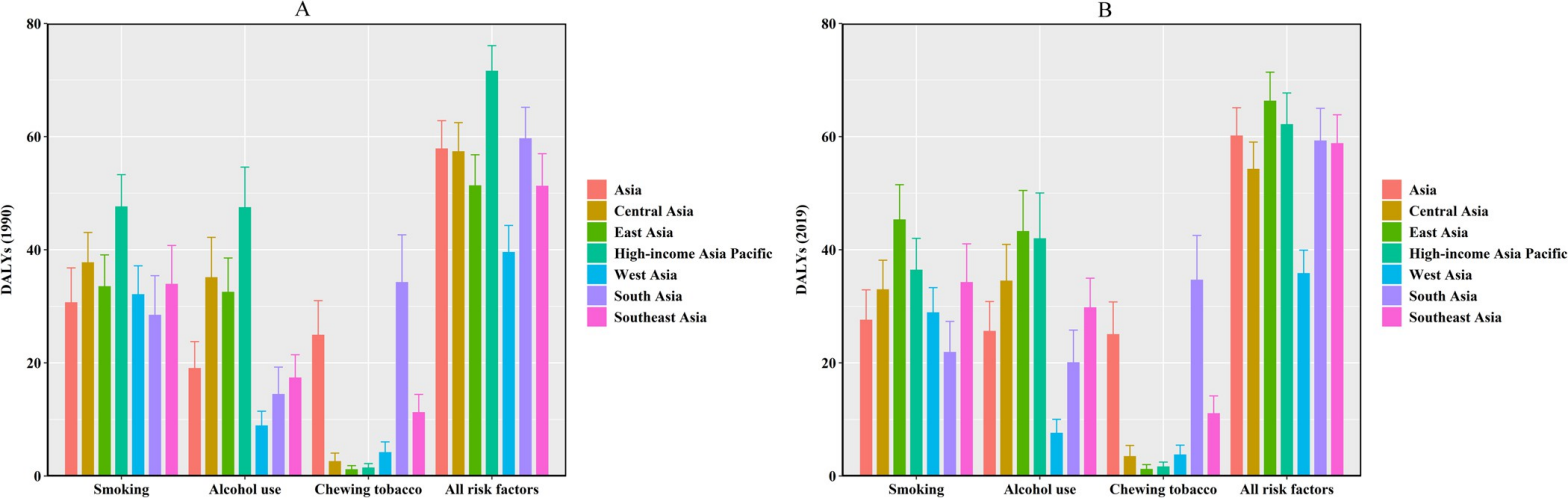
Risk factors attributable to OC burden in Asia in 1990 and 2019. (A) 1990, (B) 2019.

**Fig 7:**
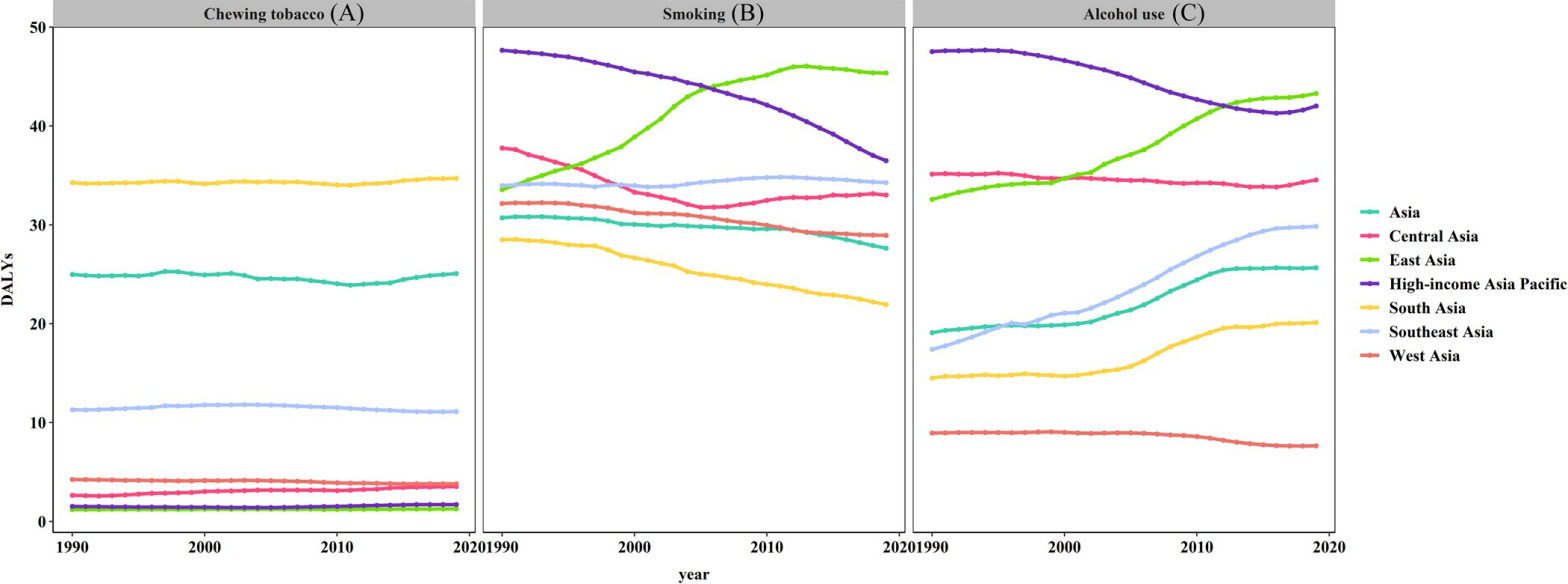
Chewing tobacco, smoking, and alcohol use contributed to DALYs across Asia from 1990 to 2019. (A) Chewing tobacco, (B) Smoking, (C) Alcohol use.

Of the 39548.26 (95% UI = 35003.48–44708.67) Asian oral cancer DALYs, 25.08% (95% UI = 19.36–30.78%) was attributable to chewing tobacco. Related oral cancer DALYs remained stable from 1990 to 2019 in Asia. The influence of chewing tobacco on DALY was most obvious in South Asia (Table 1, Figs 6A, 6B and 7A).

Smoking was the main cause of oral cancer DALYs in Asia. Smoking-related oral cancer DALY showed a downward trend from 1990 to 2019, and smoking caused 27.65% (95% UI = 21.62–32.90%) of oral cancer DALYs in 2019. However, smoking-induced oral cancer DALYs in East Asia were on the rise due to smoking from 1990 to 2019 (Fig 7B). Smoking contributed the most DALYs in 1990 in high-income Asia Pacific and in 2019 in East Asia (Table 1, Fig 6B).

Alcohol use was another main cause of oral cancer DALYs in Asia. In 2019, 25.66% of oral cancer DALYs were attributable to alcohol use (95% UI = 19.95–30.84%) with an obvious increase in Asia. Alcohol-use-related OC DALYs in Asian regions were on the rise except for the high-income Asia Pacific region and West Asia (Fig 7C).

## Discussion

To the best of our knowledge, this study has been the first to disclose the most up-to-date trends and patterns of the incidence, mortality, and DALYs of OC from 1990 to 2019 based on the GBD 2019 results. The growth rate of incidence and death rates and DALYs far exceeded the EAPC of ASIR, ASDR, and age-standardised DALYs. This discrepancy may be attributed to population growth [14]. ASIR and ASDR in males were nearly twice that in females. This gender difference in OC can be related to the frequent exposure of males to high amounts of exogenous carcinogens.

Overall, from 1990 to 2019, age-standardised DALY rates in Asia showed a declining trend. However, the trend of age-standardised DALY rates in East Asia was on the rise, which may be related to the following reasons. On the one hand, all risk factors for OC DALY remained almost stable from 1990 to 2019 in Asia (Fig 6). On the other hand, OC DALY caused by smoking and alcohol use, had been increasing from 1990 to 2019 in East Asia (Fig 7).

The incidence and prevalence of OC varied by country, and a certain geographical centralization trend was observed. The highest ASIR, ASDR, and age-standardised DALY were found in South Asia and the lowest in West Asia. Betel quid chewing and cigarette smoking were the main causes of the high incidence of OC in South Asia countries [15–17]. Chewing tobacco was an important cause of the increased burden of oral cancer in South Asia. Chewing tobacco was almost twice as common as smoking. Although the amount of nicotine in chewing tobacco was much less than that found in smoking tobacco, it may have a greater carcinogenic potential due to longer contact with oral mucous membranes. Tobacco-specific N-nitrosamines had a higher concentration in smokeless tobacco and played a key role in the malignant transformation of the oral cavity [18]. Alcohol consumption contributed to the burden of oral cancer in developing countries, such as South Asia and Southeast Asia countries. Globally, alcohol consumption had increased over the past 30 years, with more increases in low—and middle-income countries than in high-income countries [19]. In most cases, the incidence of OC in Arab countries was below the world standard rate [20]. Risk factors for oral cancer, such as smoking and alcohol consumption, were prohibited under Islamic law in predominantly Islamic Arab countries [21]. This may be the reason for the lowest ASIR, ASDR, and age-standardised of DALY in West Asia.

The three countries and territories with the highest increase number in OC incidence, deaths, and DALYs from 1990 to 2019 were Qatar, UAE, and Taiwan China. On the one hand, population growth is a factor in the rapidly increasing burden of OC in Qatar and UAE. From 1990 to 2019, the total population increased from 476,000 to 2,832,000 in Qatar and from 1,828,000 to 9,771,000 in UAE [22]. Qatar and UAE are countries with a large number of immigrants. From 1990 to 2019, the percentage of international migrants in the total population increased from 65.0% to 78.7% in Qatar and from 71.5% to 87.9% in UAE. More than 50% of international immigrants come from South Asia, such as India, Sri Lanka, Bangladesh, and other countries [23]. On the other hand, the rapid increase in the burden of oral cancer in Taiwan China is due to the epidemic of betel chewing [24].

In terms of ASIR, ASDR, and age-standardised DALY, the three countries with the most significant downward trend are Mongolia, Singapore, and Bahrain from 1990 to 2019 (Fig 4). Economic development was significantly associated with OC in each country. The incidence of OC was closely related to GDP per capita (at purchasing power parity, PPP), with a negative correlation in countries with GDP per capita (PPP) above US $10,000 [25]. Based on the World Bank database, from 1990 to 2019, Singapore’s GDP per capita rose from $37,348.2 to $97,341.5 (PPP), while Bahrain’s rose from $38,613 to $45,025.8 (PPP). In Mongolia, the decreasing burden of oral cancer from 1990 to 2019 may be related to health insurance policy. The medical insurance funds cover 85% - 90% of hospitalization expenses in public hospitals and nursing homes, and consultation services for general practitioners are free [26].

From 1990 to 2019, the ASIR of OC in the high-income Asia Pacific was on the rise, but the ASDR and age-standardised DALY decreased significantly. This opposite trend can be attributed to the increase in survival rates in high-income countries, as well as improved treatments and advances in early diagnosis at all ages [27]. Socioeconomic status was strongly associated with oral cancer risk [28]. Studies conducted in developing countries had found that most oral cancers were diagnosed in advanced stages, while in developed countries, the most prevalent stages of oral cancer are stages I and II. This result suggests that socioeconomic factors have a strong influence on delayed diagnosis and lead to a lower burden of OC in high-income countries [29].

The highest incidence and mortality rates and DALYs of OC were found among people aged 50–69 years. Many studies reported oral cancer in patients aged 50 and 69 years [30, 31]. In Saudi Arabia, 75% of oral cancers occur in people over the age of 50[32]. The average age of Americans diagnosed with oral cancer is 62 years, and two-thirds of Americans are over 55 years old [33]. In Beijing, the average age of males with OC is 63, whereas that of females with OC is 67 [34]. The risk of developing oral cancer increases with age likely because of prolonged exposure to various complex environments and risk behavior factors. Furthermore, cancer-causing genetic maturations and epigenetic modifications also increase with age. The key to reducing the role of aging in cancer is to protect people from these risk factors.

From 1990 to 2019, in the age distribution ratio, the incidence and mortality rates and DALYs of people over 70 years old increased yearly, and this increase was more pronounced in females. On the one hand, the advent of an aging society and rising life expectancy were observed. On the other hand, a long life expectancy for women indicated more time after menopause, long exposure to the risk of estrogen deficiency, and ultimately a higher risk of OC. The higher age peak of women with OC is related to estrogen deficiency [34].

Pakistan, Taiwan China, and India had the highest ASRs of incidence, death, DALYs and carried the heaviest burden of OC. Treatment of oral cancer was expensive and imposed a heavy burden on the country, health care system, and family. Iran’s economic burden of oral cancer in 2014 was $64,245,173, half of which (50%) was attributed to productivity losses. Low survival rates for OC lead to premature death and productivity losses. The cost of advanced oral cancer was five times higher than in the early stages ($10,532 vs. $2,225) [35]. The cost of OC per person in New Zealand was NZ$22,694 [36]. In Sri Lanka, the average cost of treating a stage II OC patient was SLR 136,628. Indirect and direct household expenditures were higher than the health system cost. The average cost of advanced OC was higher than that of a stage II patient, reaching SLR 375,551[10]. Due to the high cost associated with advanced oral cancer, governments should allocate resources and policy support to the early diagnosis of OC for them to be able to minimize the burden on the country, health care system, and household.

The early detection of OC led to reduced aggressive treatment and improved quality of life and overall survival. An organised, population-based oral cancer screening program targeting high-risk groups can reduce mortality in patients with stage III or IV OC. Oral cancer screening was associated with a 21% reduction in the incidence of advanced oral cancer and a 13% improvement in survival compared with non-screened populations [37]. Interestingly, artificial intelligence (AI) plays an important role in the early diagnosis of oral cancer, independent of the experience of dentists and public awareness of OC. Deep learning algorithms for the detection of the oral cavity squamous cell carcinoma from photographic images can capture fine-grained visual patterns of OC in a cluttered oral image background with a speed and reliability matching or even beyond the capabilities of human experts validate that such a non-invasive, rapid and easy-to-use tool has significant clinical implications for the early diagnosis or screening of suspected patients in countries that lack medical expertise [38]. It is believed that with the development of AI science and the popularization in the medical field, the early detection and diagnosis of OC will help to reduce the burden on the families and countries of the patients.

## Conclusion

Although the burden of OC was declining in Asia, South Asia remained the region with the highest burden. OC caused the greatest burden in Pakistan, Taiwan China and India. Therefore, measures should be taken to reduce the burden of the disease in high-risk regions and countries with attributable risk factors.

## Supporting information

S1 Table The incident cases and age-standardised incidence rate of Oral cancer in 1990 and 2019, and its temporal trends from 1990 to 2019 by gender. (DOC)

S2 Table Three countries with the largest and lowest number of incidence, death, or DALY. (DOC)

S3 Table The death cases and age-standardised death rate of oral cancer in 1990 and 2017, and its temporal trends from 1990 to 2019 by gender. (DOC)

S4 Table The incident cases and age-standardised of DALY rate of Oral cancer in 1990 and 2019, and its temporal trends from 1990 to 2019 by gender. (DOC)
