# Supplementary material for: Burden of oral cancer in Asia from 1990 to 2019: Estimates from the Global Burden of Disease 2019 study
Source: PLoS One. 2022 Mar 24;17(3):e0265950. doi: 10.1371/journal.pone.0265950 (PMC8947401; doi:10.1371/journal.pone.0265950)
Supplement: S1 Table — (DOC) [file pone.0265950.s001.doc]

**Supplementary Table 1. The incident cases and age-standardized incidence rate of Oral cancer in 1990 and 2019, and its temporal trends from 1990 to 2019.**

| Nation | sex | Incident Cases No.*10^2^  (95%UI) | | Change in absolute number(%) | ASIR per 100,000 No.(95%UI) | | 1990-2019 EAPC No.(95%CI) |
| --- | --- | --- | --- | --- | --- | --- | --- |
|  |  | 1990 | 2019 |  | 1990 | 2019 |  |
| Over all  Asia | Both | 864.13(791.30-947.36) | 2397.59(2119.66-2674.51) | 177.46% | 3.98(3.62-4.36) | 4.90(4.34-5.45) | 0.32(0.19,0.46) |
| East Asia | Both | 138.59(123.36,155.31) | 520.42(442.68,610.49) | 275.51 | 1.50(1.34,1.67) | 2.5(2.13,2.92) | 2.40(2.13,2.67) |
| Southeast Asia | Both | 111.33(100.2,120.72) | 289.54(241.63,346.54) | 160.07 | 4.13(3.70,4.49) | 4.7(3.94,5.65) | 0.14(0.1,0.18) |
| Central Asia | Both | 13.50(12.56,15.21) | 24.08(21.84,26.73) | 78.37 | 2.74(2.54,3.11) | 3.06(2.78,3.40) | 0.46(0.16,0.77) |
| High-income Asia Pacific | Both | 35.66(34.4,36.67) | 84.85(72.07,96.88) | 137.94 | 1.78(1.71,1.84) | 2.07(1.79,2.36) | 0.24(0.21,0.27) |
| South Asia | Both | 542.16(479.74,610.86) | 1432.02(1208.53,1661.69) | 164.13 | 8.82(7.71,10.01) | 9.65(8.17,11.15) | 0.18(0.09,0.26) |
| West Asia | Both | 24.17(20.3,27.88) | 66.45(58.07,76.9) | 174.93 | 1.33(1.12,1.54) | 1.46(1.28,1.68) | 0.22(0.13,0.31) |
| Armenia | Both | 0.60(0.54-0.68) | 0.81(0.66-0.96) | 35.00% | 2.08(1.87-2.33) | 1.95(1.61-2.32) | -0.26(-0.56,0.03) |
| Afghanistan | Both | 1.17(0.67-1.80) | 2.11(1.26-2.93) | 80.34% | 1.61(0.95-2.42) | 1.44(0.90-1.94) | -0.43(-0.51,-0.36) |
| Azerbaijan | Both | 0.60(0.52-0.70) | 1.58(1.25-1.97) | 163.33% | 1.13(0.97-1.33) | 1.66(1.32-2.08) | 1.85(1.45,2.24) |
| Bahrain | Both | 0.06(0.05-0.07) | 0.21(0.16-0.28) | 250.00% | 3.00(2.49-3.55) | 2.02(1.54-2.55) | -1.82(-2.05,-1.59) |
| Bangladesh | Both | 42.44(30.96-54.70) | 82.17(55.96-115.84) | 93.61% | 8.37(6.09-10.86) | 6.12(4.19-8.55) | -1.20(-1.36,-1.04) |
| Bhutan | Both | 0.20(0.13-0.27) | 0.43(0.30-0.59) | 115.00% | 7.15(4.85-9.53) | 7.36(5.22-9.72) | 0.03(-0.12,0.17) |
| Brunei Darussalam | Both | 0.07(0.06-0.08) | 0.16(0.14-0.19) | 128.57% | 6.50(5.37-7.63) | 5.45(4.74-6.22) | -0.41(-0.63,-0.19) |
| Cambodia | Both | 1.71(127-2.20) | 5.51(4.04-7.18) | 222.22% | 3.54(2.68-4.56) | 4.50(3.34-5.79) | 0.80(0.69,0.91) |
| China | Both | 123.90(108.67-140.57) | 452.16(376.90-541.79) | 264.94% | 1.40(1.23-1.57) | 2.25(1.89-2.68) | 2.33(2.01,2.63) |
| Cyprus | Both | 0.23(0.20-0.26) | 0.56(0.48-0.66) | 143.48% | 2.87(2.49-3.26) | 3.02(2.58-3.54) | 0.24(0.11,0.37) |
| Democratic People's Republic of Korea | Both | 4.25(3.15-5.64) | 7.53(5.77-9.95) | 77.18% | 2.40(1.81-3.14) | 2.32(1.78-3.03) | -0.05(-0.22,0.12) |
| Georgia | Both | 1.62(1.35-1.95) | 2.00(1.66-2.39) | 23.46% | 2.59(2.18-3.11) | 3.56(2.95-4.25) | 1.64(1.11,2.18) |
| India | Both | 390.65(344.07-441.79) | 1048.38(861.84-1247.04) | 168.37% | 7.93(6.94-9.02) | 8.82(7.22-10.44) | 0.23(0.12,0.33) |
| Indonesia | Both | 28.70(24.11-34.17) | 73.06(53.16-98.76) | 154.56% | 2.72(2.28-3.18) | 3.38(2.48-4.55) | 0.68(0.64,0.72) |
| Iran(Islamic Republic of) | Both | 2.96(2.47-3.46) | 9.29(8.51-10.20) | 213.85% | 1.08(0.88-1.27) | 1.26(1.15-1.38) | 0.37(0.24,0.5) |
| Iraq | Both | 1.36(1.09-1.66) | 4.18(3.14-5.41) | 207.35% | 1.60(1.28-1.96) | 1.63(1.25-2.04) | -0.04(-0.11,0.04) |
| Israel | Both | 0.93(0.86-1.01) | 2.58(2.01-3.27) | 177.42% | 1.94(1.79-2.10) | 2.32(1.80-2.95) | 0.26(0.01,0.51) |
| Japan | Both | 30.70(29.52-31.65) | 69.45(56.88-80.73) | 126.22% | 1.85(1.78-1.91) | 2.24(1.88-2.61) | 0.68(0.4,0.95) |
| Jordan | Both | 0.30(0.25-0.36) | 1.28(1.05-1.56) | 326.67% | 2.00(1.64-2.40) | 1.84(1.52-2.24) | -0.44(-0.53,-0.35) |
| Kazakhstan | Both | 5.36(4.89-6.37) | 6.99(5.97-8.18) | 30.41% | 4.04(3.69-4.80) | 3.89(3.33-4.52) | -0.37(-0.56,-0.17) |
| Kuwait | Both | 0.15(0.13-0.16) | 0.38(0.31-0.46) | 153.33% | 2.01(1.78-2.24) | 1.36(1.12-1.65) | -0.75(-1.07,-0.43) |
| Kyrgyzstan | Both | 1.06(0.94-1.19) | 1.22(1.03-1.43) | 15.09% | 3.35(2.99-3.77) | 2.50(2.14-2.93) | -0.67(-0.92,-0.43) |
| Lao People's Democratic Republic | Both | 0.84(0.57-1.12) | 1.56(1.17-2.02) | 85.71% | 3.75(2.58-5.00) | 3.41(2.56-4.37) | -0.54(-0.69,-0.38) |
| Lebanon | Both | 0.57(0.43-0.71) | 1.42(1.10-1.87) | 149.12% | 2.38(1.83-2.95) | 2.72(2.11-3.59) | 0.80(0.58,1.04) |
| Malaysia | Both | 4.97(4.40-5.61) | 14.44(11.18-18.47) | 190.54% | 5.06(4.45-5.75) | 5.26(4.12-6.69) | -0.36(-0.66,-0.05) |
| Maldives | Both | 0.05(0.04-0.06) | 0.16(0.13-0.19) | 220.00% | 5.89(4.54-7.01) | 5.36(4.35-6.53) | -0.75(-0.90,-0.60) |
| Mongolia | Both | 0.72(0.60-0.87) | 0.85(0.65-1.12) | 18.06% | 6.57(5.44-7.93) | 3.45(2.66-4.43) | -3.33(-3.85,-2.81) |
| Myanmar | Both | 8.58(6.03-11.54) | 16.23(12.31-21.67) | 89.16% | 3.47(2.47-4.58) | 3.45(2.65-4.57) | -0.12(-0.18,-0.06) |
| Nepal | Both | 6.60(4.67-8.75) | 15.24(11.66-19.06) | 130.91% | 6.37(4.45-8.45) | 6.65(5.13-8.20) | 0.14(-0.15,0.43) |
| Pakistan | Both | 102.26(87.03-120.32) | 285.79(229.07-359.35) | 179.47% | 16.60(14.02-19.55) | 21.93(17.83-27.56) | 0.85(0.66,1.04) |
| Palestine | Both | 0.12(0.09-0.16) | 0.32(0.27-0.38) | 166.67% | 1.30(0.93-1.75) | 1.28(1.07-1.50) | -0.19(-0.40,0.03) |
| Philippines | Both | 14.64(12.95-16.26) | 27.80(22.39-34.48) | 89.89% | 4.50(3.97-5.01) | 3.45(2.80-4.23) | -1.38(-1.63,-1.13) |
| Qatar | Both | 0.03(0.02-0.03) | 0.22(0.16-0.29) | 633.33% | 2.23(1.70-2.84) | 2.30(1.77-2.93) | 0.47(0.19,0.74) |
| Republic of Korea | Both | 4.24(4.00-4.54) | 14.02(11.51-16.83) | 230.66% | 1.35(1.27-1.45) | 1.62(1.33-1.94) | 0.01(-0.56,0.58) |
| Saudi Arabia | Both | 0.95(0.70-1.18) | 4.84(3.62-6.46) | 409.47% | 1.47(1.09-1.81) | 2.09(1.64-2.66) | 1.30(1.22,1.38) |
| Singapore | Both | 0.65(0.61-0.70) | 1.21(0.97-1.53) | 86.15% | 2.78(2.59-2.98) | 1.55(1.24-1.95) | -1.87(-2.06,-1.70) |
| Sri Lanka | Both | 7.28(6.51-8.14) | 20.69(15.05-27.31) | 184.20% | 6.74(6.04-7.50) | 8.12(5.94-0.63) | 0.52(0.29,0.76) |
| Taiwan China | Both | 10.44(9.86-11.06) | 60.72(46.19-80.03) | 481.61% | 5.89(5.56-6.24) | 16.35(12.42-21.52) | 3.75(3.12,4.37) |
| Tajikistan | Both | 0.39(0.30-0.51) | 0.72(0.57-0.91) | 84.62% | 1.35(1.01-1.83) | 1.47(1.19-1.82) | 0.33(0.08,0.58) |
| Thailand | Both | 23.17(20.30-26.40) | 57.23(42.02-75.55) | 147.00% | 6.30(5.53-7.21) | 5.72(4.21-7.55) | -0.92(-1.13,-0.70) |
| Timor-Leste | Both | 0.08(0.06-0.10) | 0.25(0.18-0.33) | 212.50% | 2.42(1.85-3.11) | 3.06(2.25-3.90) | 0.93(0.64,1.23) |
| Turkmenistan | Both | 0.67(0.62-0.72) | 1.52(1.21-1.93) | 126.87% | 3.23(3.00-3.48) | 3.61(2.86-4.54) | 0.36(0.11,0.61) |
| United Arab Emirates | Both | 0.19(0.12-0.27) | 1.58(0.90-2.79) | 731.58% | 2.88(1.57-4.17) | 2.49(1.53-4.11) | -0.74(-1.01,-0.47) |
| Uzbekistan | Both | 2.49(2.07-3.36) | 8.38(6.94-10.01) | 236.55% | 1.97(1.62-2.74) | 3.53(2.96-4.12) | 1.88(1.55,2.19) |
| Viet Nam | Both | 20.78(16.57-25.85) | 71.18(53.94-90.73) | 242.54% | 4.97(3.97-6.12) | 7.16(5.50-9.06) | 1.26(1.21,1.30) |
| Afghanistan | Female | 1.17(0.67-1.80) | 0.92(0.57-1.60) | -21.37% | 1.08(0.76-1.64) | 1.18(0.77-1.88) | 0.29(0.20,0.38) |
| Armenia | Female | 0.18(0.15-0.21) | 0.22(0.18-0.26) | 22.22% | 1.14(0.98-1.35) | 0.97(0.80-1.17) | -0.58(-0.83,-0.32) |
| Azerbaijan | Female | 0.26(0.21-0.30) | 0.63(0.49-0.80) | 142.31% | 0.84(0.70-1.00) | 1.23(0.96-1.58) | 1.66(1.39,1.93) |
| Bahrain | Female | 0.06(0.05-0.07) | 0.05(0.04-0.07) | -16.67% | 1.96(1.64-2.32) | 1.38(1.10-1.71) | -1.67(-1.97,-1.37) |
| Bangladesh | Female | 11.55(8.74-14.15) | 33.04(21.78-44.69) | 186.06% | 4.95(3.76-6.19) | 5.05(3.32-6.78) | -0.05(-0.18,0.09) |
| Bhutan | Female | 0.07(0.04-0.09) | 0.17(0.12-0.22) | 142.86% | 4.92(3.47-6.38) | 5.97(4.33-7.66) | 0.53(0.42,0.64) |
| Brunei Darussalam | Female | 0.02(0.02-0.03) | 0.07(0.05-0.08) | 250.00% | 3.82(3.28-4.47) | 4.17(3.55-4.90) | 0.31(0.16,0.45) |
| Cambodia | Female | 0.71(0.53-0.96) | 2.27(1.61-3.16) | 219.72% | 2.62(2.00-3.44) | 3.29(2.37-4.52) | 0.71(0.65,0.78) |
| China | Female | 53.35(44.32-62.97) | 117.37(94.74-144.81) | 120.00% | 1.17(0.98-1.37) | 1.16(0.94-1.43) | -0.11(-0.19,-0.02) |
| Cyprus | Female | 0.23(0.20-0.26) | 0.21(0.17-0.25) | -8.69% | 1.80(1.53-2.09) | 2.14(1.75-2.57) | 0.77(0.64,0.90) |
| Democratic People's Republic of Korea | Female | 1.92(1.48-2.47) | 3.00(2.31-3.85) | 56.25% | 1.90(1.46-2.42) | 1.67(1.28-2.15) | -0.44(-0.64,-0.24) |
| Georgia | Female | 0.39(0.31-0.47) | 0.39(0.32-0.46) | 0.00% | 1.08(0.86-1.30) | 1.16(0.95-1.39) | 0.51(0.24,0.78) |
| India | Female | 123.47(100.51-148.42) | 394.40(312.73-486.34) | 219.43% | 5.26(4.25-6.37) | 6.64(5.27-8.16) | 0.58(0.42,0.74) |
| Indonesia | Female | 13.1263(9.99-18.28) | 30.79(21.11-47.45) | 134.57% | 2.43(1.88-3.28) | 2.79(1.93-4.27) | 0.31(0.24,0.39) |
| Iran(Islamic Republic of) | Female | 2.96(2.47-3.46) | 4.29(3.89-4.78) | 44.93% | 0.79(0.66-0.90) | 1.16(1.05-1.30) | 1.24(1.09,1.40) |
| Iraq | Female | 1.36(1.09-1.66) | 2.06(1.52-2.74) | 51.47% | 1.23(0.94-1.56) | 1.54(1.15-2.01) | 0.8(0.73,0.87) |
| Israel | Female | 0.93(0.86-1.01) | 0.98(0.76-1.25) | 5.38% | 1.45(1.30-1.61) | 1.60(1.23-2.05) | 0.02(-0.19,0.23) |
| Japan | Female | 10.95(10.19-11.50) | 29.34(22.37-36.09) | 167.95% | 1.20(1.12-1.26) | 1.57(1.24-1.93) | 0.94(0.67,1.21) |
| Jordan | Female | 0.30(0.25-0.36) | 0.44(0.34-0.58) | 46.67% | 1.42(1.13-1.73) | 1.35(1.06-1.72) | -0.51(-0.72,-0.30) |
| Kazakhstan | Female | 1.58(1.39-1.84) | 2.56(2.14-3.12) | 62.40% | 2.02(1.79-2.37) | 2.50(2.10-3.04) | 0.76(0.44,1.08) |
| Kuwait | Female | 0.15(0.13-0.16) | 0.15(0.11-0.20) | 0.00% | 1.72(1.47-1.97) | 1.22(0.94-1.61) | -0.27(-0.77,0.23) |
| Kyrgyzstan | Female | 0.29(0.25-0.32) | 0.42(0.35-0.52) | 44.83% | 1.57(1.38-1.76) | 1.59(1.32-1.93) | 0.22(0.01,0.44) |
| Lao People's Democratic Republic | Female | 0.30(0.19-0.47) | 0.61(0.44-0.87) | 103.33% | 2.60(1.73-3.87) | 2.61(1.91-3.66) | -0.23(-0.37,-0.09) |
| Lebanon | Female | 0.57(0.43-0.71) | 0.59(0.45-0.79) | 3.51% | 1.55(1.27-1.92) | 2.07(1.59-2.76) | 1.11(1.06,1.17) |
| Malaysia | Female | 2.47(2.15-2.79) | 7.29(5.63-9.40) | 194.99% | 4.99(4.33-5.70) | 5.41(4.19-6.92) | -0.05(-0.28,0.17) |
| Maldives | Female | 0.03(0.02-0.04) | 0.09(0.08-0.12) | 214.67% | 7.81(5.71-11.07) | 7.23(5.77-8.84) | -0.64(-0.79,-0.48) |
| Mongolia | Female | 0.34(0.27-0.41) | 0.29(0.227-0.38) | -14.88% | 5.41(4.38-6.59) | 2.23(1.69-2.92) | -4.34(-4.97,-3.71) |
| Myanmar | Female | 3.59(2.45-5.60) | 7.07(5.14-10.35) | 97.06% | 2.76(1.93-4.15) | 2.74(2.02-4.01) | -0.20(-0.28,-0.12) |
| Nepal | Female | 2.40(1.85-3.05) | 6.81(4.98-8.75) | 183.78% | 4.75(3.62-6.06) | 5.64(4.16-7.13) | 0.53(0.34,0.71) |
| Pakistan | Female | 41.80(33.92-51.56) | 132.29(99.84-174.40) | 216.49% | 14.74(11.8-18.27) | 20.86(16.04-27.08) | 1.15(1.02,1.28) |
| Palestine | Female | 0.12(0.09-0.16) | 0.15(0.12-0.18) | 25.00% | 0.96(0.71-1.25) | 1.14(0.93-1.37) | 0.47(0.25,0.70) |
| Philippines | Female | 6.45(5.50-7.46) | 11.87(8.97-15.23) | 83.96% | 4.04(3.44-4.65) | 2.85(2.17-3.62) | -1.69(-1.93,-1.43) |
| Qatar | Female | 0.03(0.02-0.03) | 0.07(0.05-0.09) | 133.33% | 2.26(1.59-3.07) | 3.4(2.64-4.33) | 2.02(1.67,2.36) |
| Republic of Korea | Female | 1.36(1.23-1.53) | 5.03(4.05-6.11) | 269.93% | 0.79(0.71-0.88) | 1.09(0.88-1.32) | 0.55(0.08,1.03) |
| Saudi Arabia | Female | 0.95(0.70-1.18) | 2.27(1.69-3.04) | 138.95% | 1.31(0.95-1.72) | 2.43(1.88-3.14) | 2.35(2.22,2.47) |
| Singapore | Female | 0.24(0.21-0.26) | 0.46(0.36-0.58) | 90.79% | 1.85(1.67-2.04) | 1.16(0.90-1.47) | -1.56(-1.75,-1.37) |
| Sri Lanka | Female | 2.98(2.52-3.47) | 7.58(5.64-10.13) | 154.27% | 5.52(4.68-6.45) | 5.41(4.03-7.24) | -0.71(-1.03,-0.38) |
| Taiwan China | Female | 1.82(1.66-2.00) | 7.11(5.41-9.50) | 290.68% | 2.27(2.09-2.48) | 3.62(2.73-4.82) | 1.86(1.68,2.04) |
| Tajikistan | Female | 0.18(0.13-0.22) | 0.35(0.27-0.45) | 94.83% | 1.11(0.81-1.38) | 1.30(1.03-1.63) | 0.52(0.37,0.67) |
| Thailand | Female | 9.80(8.28-11.43) | 25.37(18.84-33.28) | 158.91% | 5.24(4.41-6.13) | 4.70(3.51-6.18) | -0.94(-1.22,-0.65) |
| Timor-Leste | Female | 0.03(0.03-0.04) | 0.11(0.08-0.15) | 270.67% | 2.25(1.76-2.83) | 2.70(2.06-3.52) | 0.66(0.43,0.89) |
| Turkmenistan | Female | 0.21(0.19-0.24) | 0.59(0.45-0.75) | 179.86% | 1.78(1.61-2.00) | 2.58(2.02-3.24) | 1.76(1.40,2.12) |
| United Arab Emirates | Female | 0.19(0.12-0.27) | 0.24(0.12-0.48) | 26.32% | 1.58(0.86-2.92) | 1.66(0.92-3.18) | 0.26(-0.34,0.85) |
| Uzbekistan | Female | 1.11(0.86-1.65) | 3.61(2.88-4.38) | 224.86% | 1.56(1.20-2.35) | 2.77(2.27-3.32) | 1.86(1.59,2.12) |
| Viet Nam | Female | 8.66(6.82-10.76) | 21.95(16.81-28.13) | 153.45% | 3.64(2.88-4.51) | 4.13(3.17-5.28) | 0.29(0.18,0.40) |
| Afghanistan | Male | 0.79(0.38-1.42) | 1.18(0.65-2.00) | 49.37% | 2.10(1.05-3.71) | 1.72(1.01-2.77) | -0.71(-0.76,-0.66) |
| Armenia | Male | 0.42(0.36-0.49) | 0.59(0.48-0.71) | 40.48% | 3.34(2.89-3.85) | 3.23(2.64-3.89) | -0.17(-0.50,0.15) |
| Azerbaijan | Male | 0.34(0.27-0.42) | 0.95(0.68-1.31) | 179.41% | 1.56(1.25-1.96) | 2.22(1.63-3.01) | 1.88(1.37,2.39) |
| Bahrain | Male | 0.04(0.03-0.05) | 0.16(0.11-0.22) | 300.00% | 3.88(3.10-4.77) | 2.47(1.82-3.23) | -1.98(-2.33,-1.64) |
| Bangladesh | Male | 30.90(20.31-42.84) | 49.13(33.49-76.76) | 59.00% | 11.18(7.43-15.42) | 7.12(4.89-11.12) | -1.69(-1.86,-1.50) |
| Bhutan | Male | 0.13(0.08-0.19) | 0.26(0.17-0.39) | 100.00% | 9.42(5.60-13.30) | 8.67(5.76-12.58) | -0.32(-0.51,-0.12) |
| Brunei Darussalam | Male | 0.05(0.04-0.06) | 0.10(0.08-0.12) | 100.00% | 9.75(7.45-12.01) | 7.31(6.00-8.87) | -0.66(-1.00,-0.31) |
| Cambodia | Male | 1.00(0.67-1.50) | 3.24(2.33-4.35) | 224.00% | 4.74(3.21-7.04) | 6.18(4.45-8.30) | 0.92(0.79,1.06) |
| China | Male | 70.55(57.37-84.25) | 334.79(261.52-420.11) | 374.54% | 1.68(1.38-1.99) | 3.48(2.75-4.31) | 3.57(3.14,4.01) |
| Cyprus | Male | 0.15(0.13-0.18) | 0.35(0.28-0.44) | 133.33% | 4.05(3.34-4.84) | 3.95(3.14-4.87) | -0.10(-0.24,0.04) |
| Democratic People's Republic of Korea | Male | 2.33(1.54-3.39) | 4.53(3.18-6.49) | 94.42% | 3.16(2.19-4.48) | 3.18(2.32-4.38) | 0.14(0.01,0.27) |
| Georgia | Male | 1.23(1.01-1.54) | 1.61(1.32-1.93) | 30.89% | 4.72(3.92-5.87) | 6.68(5.51-8.00) | 1.86(1.2,2.53) |
| India | Male | 267.18(219.49-323.61) | 653.98(502.92-818.29) | 144.77% | 10.41(8.43-12.60) | 11.04(8.53-13.77) | 0.12(0.02,0.21) |
| Indonesia | Male | 15.58(12.62-18.61) | 42.26(29.86-58.20) | 171.25% | 3.04(2.48-3.59) | 4.00(2.87-5.42) | 0.98(0.92,1.05) |
| Iran(Islamic Republic of) | Male | 1.91(1.51-2.35) | 5.00(4.47-5.60) | 161.78% | 1.37(1.08-1.68) | 1.36(1.21-1.52) | -0.20(-0.32,-0.09) |
| Iraq | Male | 0.82(0.61-1.06) | 2.12(1.56-2.78) | 158.54% | 1.97(1.49-2.54) | 1.74(1.32-2.22) | -0.66(-0.77,-0.56) |
| Israel | Male | 0.56(0.49-0.62) | 1.60(1.22-2.07) | 185.71% | 2.51(2.23-2.79) | 3.13(2.39-4.06) | 0.36(0.06,0.65) |
| Japan | Male | 19.75(19.07-20.43) | 40.11(32.64-49.29) | 103.09% | 2.64(2.54-2.73) | 2.96(2.40-3.64) | 0.43(0.16,0.70) |
| Jordan | Male | 0.20(0.15-0.25) | 0.84(0.62-1.11) | 320.00% | 2.57(2.02-3.22) | 2.28(1.71-2.97) | -0.43(-0.58,-0.29) |
| Kazakhstan | Male | 3.78(3.38-4.66) | 4.43(3.70-5.23) | 17.20% | 7.24(6.47-8.89) | 5.95(5.02-6.98) | -0.97(-1.13,-0.79) |
| Kuwait | Male | 0.10(0.09-0.11) | 0.23(0.18-0.30) | 130.00% | 2.17(1.87-2.50) | 1.45(1.12-1.85) | -0.99(-1.31,-0.66) |
| Kyrgyzstan | Male | 0.77(0.66-0.90) | 0.79(0.65-0.95) | 2.60% | 5.87(5.13-6.82) | 3.70(3.09-4.40) | -1.17(-1.47,-0.89) |
| Lao People's Democratic Republic | Male | 0.54(0.32-0.84) | 0.95(0.67-1.26) | 75.93% | 5.02(3.04-7.73) | 4.25(3.00-5.46) | -0.76(-0.93,-0.58) |
| Lebanon | Male | 0.38(0.26-0.50) | 0.83(0.62-1.13) | 118.42% | 3.23(2.24-4.22) | 3.51(2.59-4.80) | 0.78(0.43,1.14) |
| Malaysia | Male | 2.50(2.04-3.05) | 7.15(5.28-9.55) | 186.00% | 5.13(4.19-6.21) | 5.09(3.78-6.72) | -0.68(-1.08,-0.27) |
| Maldives | Male | 0.03(0.02-0.04) | 0.06(0.05-0.08) | 140.00% | 4.75(3.24-6.68) | 3.76(2.97-4.67) | -1.37(-1.56,-1.17) |
| Mongolia | Male | 0.39(0.30-0.48) | 0.56(0.42-0.75) | 43.59% | 7.97(6.26-9.90) | 5.07(3.85-6.59) | -2.53(-2.97,-2.10) |
| Myanmar | Male | 5.00(3.21-7.71) | 9.16(6.70-12.01) | 83.20% | 4.25(2.78-6.51) | 4.34(3.18-5.63) | 0.03(-0.07,0.14) |
| Nepal | Male | 4.20(2.60-6.02) | 8.43(6.25-10.91) | 100.71% | 7.9(4.89-11.21) | 7.74(5.76-9.92) | -0.05(-0.42,0.32) |
| Pakistan | Male | 60.45(50.59-72.37) | 153.50(109.54-212.60) | 153.93% | 18.16(15.13-21.86) | 22.91(16.52-31.61) | 0.65(0.42,0.88) |
| Palestine | Male | 0.07(0.05-0.10) | 0.17(0.14-0.21) | 142.86% | 1.71(1.17-2.48) | 1.45(1.20-1.75) | -0.75(-0.94,-0.55) |
| Philippines | Male | 8.19(6.99-9.56) | 15.94(12.00-20.80) | 94.63% | 4.96(4.22-5.89) | 4.09(3.11-5.31) | -1.10(-1.35,-0.87) |
| Qatar | Male | 0.02(0.01-0.02) | 0.15(0.10-0.21) | 650.00% | 2.22(1.61-2.93) | 1.90(1.39-2.57) | -0.39(-0.68,-0.11) |
| Republic of Korea | Male | 2.88(2.66-3.13) | 8.99(7.17-11.02) | 212.15% | 2.15(1.99-2.36) | 2.24(1.81-2.72) | -0.56(-1.18,0.08) |
| Saudi Arabia | Male | 0.59(0.41-0.78) | 2.57(1.84-3.57) | 335.59% | 1.57(1.10-2.06) | 1.87(1.40-2.45) | 0.54(0.46,0.61) |
| Singapore | Male | 0.42(0.38-0.46) | 0.75(0.58-0.98) | 78.57% | 3.89(3.53-4.26) | 1.97(1.54-2.52) | -2.16(-2.38,-1.93) |
| Sri Lanka | Male | 4.31(3.75-4.95) | 13.12(8.98-17.90) | 204.41% | 7.96(6.90-9.13) | 11.47(7.92-15.50) | 1.49(1.15,1.85) |
| Taiwan China | Male | 8.61(8.07-9.21) | 53.61(40.53-71.28) | 522.65% | 9.21(8.63-9.81) | 29.87(22.62-39.49) | 4.26(3.54,4.98) |
| Tajikistan | Male | 0.21(0.15-0.32) | 0.37(0.29-0.48) | 76.19% | 1.68(1.21-2.78) | 1.68(1.33-2.12) | 0.13(-0.24,0.50) |
| Thailand | Male | 13.36(11.00-15.88) | 31.85(22.67-43.14) | 138.40% | 7.36(6.08-8.74) | 6.73(4.84-9.01) | -0.91(-1.14,-0.66) |
| Timor-Leste | Male | 0.04(0.03-0.06) | 0.14(0.089-0.20) | 233.33% | 2.58(1.55-3.72) | 3.42(2.14-4.74) | 1.19(0.83,1.54) |
| Turkmenistan | Male | 0.46(0.42-0.51) | 0.94(0.74-1.19) | 104.35% | 5.26(4.79-5.81) | 4.95(3.90-6.21) | -0.49(-0.79,-0.20) |
| United Arab Emirates | Male | 0.16(0.09-0.23) | 1.33(0.75-2.35) | 731.25% | 3.66(1.97-5.29) | 2.80(1.72-4.48) | -1.30(-1.56,-1.05) |
| Uzbekistan | Male | 1.37(1.14-1.84) | 4.78(3.95-5.78) | 248.91% | 2.53(2.10-3.58) | 4.52(3.78-5.39) | 1.87(1.50,2.22) |
| Viet Nam | Male | 12.12(9.34-15.65) | 49.23(36.33-64.84) | 306.19% | 6.69(5.18-8.56) | 10.90(8.21-14.11) | 1.76(1.73,1.78) |
