# Supplementary material for: Burden of oral cancer in Asia from 1990 to 2019: Estimates from the Global Burden of Disease 2019 study
Source: PLoS One. 2022 Mar 24;17(3):e0265950. doi: 10.1371/journal.pone.0265950 (PMC8947401; doi:10.1371/journal.pone.0265950)
Supplement: S2 Table — (DOC) [file pone.0265950.s002.doc]

Supplementary Table 2. Three countries with the largest and lowest number of incidence, death, or DALY.

| Measure | sex | |  | Top three contry |  |  | Bottom three contry |  | |
| --- | --- | --- | --- | --- | --- | --- | --- | --- | --- |
| 2019 ASR(per 100,000) |  | |  |  |  |  |  |  | |
| ASIR |  | |  |  |  |  |  |  | |
|  | both | | Pakistan (21.93) | Taiwan China (16.35) | India (8.82) | Iran (1.26) | Palestine (1.28) | Kuwait (1.36) | |
|  | female | | Pakistan (20.86) | Maldives (7.23) | India (6.64) | Armenia (0.97) | Republic of Korea (1.09) | Palestine (1.14) | |
|  | male | | Taiwan China (29.87) | Pakistan (22.91) | Sri Lanka (11.47) | Iran (1.36) | Kuwait (1.45) | Palestine (1.45) | |
| ASDR |  | |  |  |  |  |  |  | |
|  | both | | Pakistan (14.72) | Taiwan China (5.86) | India (5.81) | Iran (0.71) | Kuwait (0.72) | Palestine (0.76) | |
|  | female | | Pakistan (13.37) | India (4.27) | Bhutan (3.76) | China (0.50) | Armenia (0.52) | Republic of Korea  (0.59) | |
|  | male | | Pakistan (15.97) | Taiwan China (10.95) | India (7.4) | Kuwait (0.78) | Iran (0.80) | Palestine (0.94) | |
| Age Standardized DALY Rate |  | |  |  |  |  |  |  | |
|  | both | | Pakistan (421.87) | Taiwan China (188.97) | India (154.90) | Kuwait (15.30) | Iran (15.98) | Palestine (17.63) | |
|  | female | | Pakistan (375.37) | India (106.80) | Nepal (92.17) | China (11.94) | Armenia  (12.55) | Republic of Korea (12.61) | |
|  | male | | Pakistan (465.64) | Taiwan China (358.50) | India (203.16) | Kuwait (17.12) | Iran (18.27) | Palestine (20.96) | |
| 1990-2019 increase times | |  |  |  |  |  |  |  | |
| Incidence(cases) |  | |  |  |  |  |  |  | |
|  | both | | United Arab Emirates (731.58%) | Qatar (633.33%) | Taiwan China (481.61%) | Kyrgyzstan (15.09%) | Mongolia (18.06%) | | Georgia (23.46%) |
|  | female | | Taiwan China (290.68%) | Timor-Leste (270.67%) | Republic of Korea (269.93) | Afghanistan (-21.37%) | Bahrain (-16.67%) | Mongolia (-14.88%) | |
|  | male | | United Arab Emirates (731.25%) | Qatar (650.00%) | Taiwan China (522.65%) | Kyrgyzstan (2.60%) | Kazakhstan (17.20%) | Georgia (30.89%) | |
| Death (cases) |  | |  |  |  |  |  |  | |
|  | both | | Qatar (800.00%) | United Arab Emirates (650.00%) | Taiwan China (430.33) | Mongolia (6.00%) | Kyrgyzstan (7.35%) | Kazakhstan (18.02%) | |
|  | female | | United Arab Emirates (400.00%) | Maldives (300.00%) | Brunei Darussalam (300.00%) | Mongolia (-27.27%) | Georgia (9.09%) | Armenia (20.00%) | |
|  | male | | United Arab Emirates (712.50%) | Qatar (500.00%) | Taiwan China (456.39) | Kyrgyzstan (-1.96%) | Kazakhstan (9.13%) | Mongolia (32.14%) | |
| DALY(Years) |  | |  |  |  |  |  |  | |
|  | both | | United Arab Emirates (670.36%) | Qatar (506.12%) | Taiwan China (387.30%) | Kyrgyzstan (3.16%) | Armenia (9.68%) | Kazakhstan (12.88) | |
|  | female | | United Arab Emirates (591.07%) | Qatar (530.77%) | Saudi Arabia (308.85%) | Mongolia (-21.18%) | Georgia (-8.27%) | Armenia (-5.17%) | |
|  | male | | United Arab Emirates (683.73%) | Qatar (514.29%) | Taiwan China (412.18%) | Kyrgyzstan (-6.52%) | Kazakhstan (2.45%) | Armenia (15.03%) | |
| EAPC |  | |  |  |  |  |  |  | |
| Incidence |  | |  |  |  |  |  |  | |
|  | both | | Taiwan China (3.75) | China (2.33) | Uzbekistan (1.88) | Mongolia (-3.33) | Singapore (-1.87) | Bahrain (-1.82) | |
|  | female | | Saudi Arabia (2.35) | Qatar (2.02) | Taiwan China (1.86) | Mongolia (-4.34) | Philippines (-1.69) | Bahrain (-1.67) | |
|  | male | | Taiwan China (4.26) | China (3.57) | Azerbaijan (1.88) | Mongolia (-2.53) | Singapore (-2.16) | Bahrain (-1.98) | |
| Death |  | |  |  |  |  |  |  | |
|  | both | | Taiwan China (3.18) | Uzbekistan (1.80) | Georgia (1.79) | Mongolia (-3.63) | Bahrain (-2.37) | Singapore (-2.35) | |
|  | female | | Pakistan (1.62) | Uzbekistan (1.14) | Qatar (1.09) | Mongolia (-4.46) | Bahrain (-2.38) | Armenia (-2.02) | |
|  | male | | Taiwan China (3.73) | China (2.55) | Georgia (2.00) | Mongolia (-2.90) | Singapore (-2.59) | Bahrain (-2.44) | |
| DALY |  | |  |  |  |  |  |  | |
|  | both | | Taiwan China (3.22) | Georgia (1.68) | Uzbekistan (1.38) | Mongolia (-3.61) | Singapore (-2.66) | Bahrain (-2.66) | |
|  | female | | Turkmenistan (1.40) | Uzbekistan (1.39) | Taiwan China (1.13) | Mongolia (-4.83) | Bahrain (-2.67) | Singapore (-2.37) | |
|  | male | | Taiwan China (3.66) | China (2.42) | Georgia (1.88) | Singapore (-2.93) | Bahrain (-2.82) | Mongolia (-2.79) | |
| Cases |  | |  |  |  |  |  |  | |
| Incidence (2019)  (No. *10^2^) |  | |  |  |  |  |  |  | |
|  | both | | India  (1048.38) | China  (452.16) | Pakistan  (285.79) | Brunei Darussalam  (0.16) | Maldives  (0.16) | Bahrain  (0.21) | |
|  | female | | India  (394.40) | Pakistan  (132.29) | China  (117.37) | Bahrain  (0.05) | Brunei Darussalam  (0.07) | Qatar  (0.07) | |
|  | male | | India  (653.98) | China  (334.79) | Pakistan  (153.50) | Maldives  (0.06) | Brunei Darussalam  (0.10) | Timor-Leste  (0.14) | |
| Death |  | |  |  |  |  |  |  | |
|  | both | | India  (655.71) | China  (226.41) | Pakistan  (175.66) | Maldives  (0.07) | Brunei Darussalam  (0.09) | Qatar  (0.09) | |
|  | female | | India  (239.42) | Pakistan  (Pakistan) | China  (50.34) | Bahrain  (0.02) | Qatar  (Qatar) | Brunei Darussalam  (0.04) | |
|  | male | | India  (416.29) | China  (416.29) | Pakistan  (99.55) | Maldives  (0.03) | Brunei Darussalam  (0.06) | Qatar  (0.06) | |
| DALY |  | |  |  |  |  |  |  | |
|  | both | | India  (19226.64) | Pakistan  (5940.89) | China  (5758.05) | Maldives  (1.73) | Brunei Darussalam  (2.82) | Qatar  (2.97) | |
|  | female | | India  (6572.54) | Pakistan  (2565.86) | China  (1210.21) | Bahrain  (0.67) | Qatar  (0.82) | Maldives  (0.88) | |
|  | male | | India  (12654.1) | China  (4547.85) | Pakistan  (3375.03) | Maldives  (0.85) | Brunei Darussalam  (1.78) | Qatar  (2.15) | |
