# Supplementary material for: Burden of oral cancer in Asia from 1990 to 2019: Estimates from the Global Burden of Disease 2019 study
Source: PLoS One. 2022 Mar 24;17(3):e0265950. doi: 10.1371/journal.pone.0265950 (PMC8947401; doi:10.1371/journal.pone.0265950)
Supplement: S3 Table — (DOC) [file pone.0265950.s003.doc]

**Supplementary Table 3. The death cases and age-standardized death rate of oral cancer in 1990 and 2019, and its temporal trends from 1990 to 2019.**

| Nation | sex | Death Cases No.*10^2^  (95%UI) | | Change in absolute number(%) | ASDR per 100,000 No.(95%UI) | | 1990-2019 EAPC No.(95%CI) |
| --- | --- | --- | --- | --- | --- | --- | --- |
|  |  | 1990 | 2019 |  | 1990 | 2019 |  |
| Over all  Asia | Both | 559.80(506.20-618.28) | 1392.57(1233.11-1568.17) | 148.76% | 2.76(2.49-3.04) | 2.93(2.6-3.29) | 0.18(0.14,0.22) |
| East Asia | Both | 80.24(70.44,90.03) | 252.7(215.47,296.77) | 214.93% | 0.96(0.85,1.07) | 1.25(1.06,1.45) | 1.53(1.27,1.80) |
| Southeast Asia | Both | 64.64(58.01,69.8) | 153.25(129.11,182.71) | 137.08% | 2.65(2.36,2.86) | 2.68(2.26,3.19) | -0.05(-0.09,-0.02) |
| Central Asia | Both | 8.40(7.80,9.56) | 13.79(12.49,15.30) | 64.17% | 1.76(1.63,2.02) | 1.85(1.68,2.04) | 0.02(-0.31,0.36) |
| High-income Asia Pacific | Both | 20.86(19.96,21.40) | 55.52(47.13,60.29) | 166.16% | 1.08(1.02,1.11) | 1.15(1.02,1.23) | -0.35(-0.39,-0.31) |
| South Asia | Both | 372.67(323.24,423.52) | 893.39(769.9,1043.03) | 139.73% | 6.61(5.73,7.55) | 6.36(5.48,7.39) | -0.29(-0.39,-0.20) |
| West Asia | Both | 15.29(12.79,17.74) | 35.37(31.04,40.88) | 131.33% | 0.92(0.77,1.07) | 0.85(0.75,0.97) | -0.15(-0.23,-0.07) |
| Armenia | Both | 0.36(0.32-0.40) | 0.47(0.39-0.56) | 30.56% | 1.31(1.17-1.46) | 1.14(0.95-1.34) | -0.57(-0.86,-0.29) |
| Afghanistan | Both | 0.89(0.51-1.37) | 1.40(0.85-1.93) | 57.30% | 1.27(0.77-1.92) | 1.09(0.70-1.48) | -0.59(-0.64,0.54) |
| Azerbaijan | Both | 0.38(0.32-0.44) | 0.93(0.72-1.16) | 144.74% | 0.75(0.64-0.89) | 1.06(0.83-1.34) | 1.72(1.34,2.11) |
| Bahrain | Both | 0.03(0.03-0.04) | 0.10(0.07-0.13) | 233.33% | 2.01(1.68-2.35) | 1.14(0.88-1.43) | -2.37(-2.61,-2.17) |
| Bangladesh | Both | 30.32(22.03-39.34) | 51.86(35.79-72.17) | 71.04% | 6.37(4.63-8.20) | 4.06(2.83-5.60) | -1.59(-1.80,-1.54) |
| Bhutan | Both | 0.13(0.09-0.18) | 0.26(0.18-0.35) | 100.00% | 5.46(3.67-7.22) | 4.83(3.52-6.22) | -0.53(-0.65,-0.41) |
| Brunei Darussalam | Both | 0.04(0.04-0.05) | 0.09(0.08-0.11) | 125.00% | 4.78(3.97-5.56) | 3.79(3.32-4.31) | -0.58(-0.78,-0.37) |
| Cambodia | Both | 1.10(0.82-1.45) | 3.19(2.38-4.11) | 190.00% | 2.51(1.89-3.30) | 2.83(2.13-3.59) | 0.40(0.33,0.47) |
| China | Both | 74.03(64.37-83.57) | 226.41(189.08-270.77) | 205.84% | 0.92(0.81-1.03) | 1.16(0.98-1.38) | 1.44(1.15,1.73) |
| Cyprus | Both | 0.09(0.08-0.10) | 0.20(0.17-0.23) | 122.22% | 1.25(1.11-1.40) | 1.1(0.96-1.26) | -0.47(-0.58,0.36) |
| Democratic People's Republic of Korea | Both | 1.90(1.48-2.63) | 3.89(3.05-5.06) | 104.74% | 1.22(0.93-1.59) | 1.22(0.97-1.58) | 0.08(0.03,0.13) |
| Georgia | Both | 0.96(0.81-1.15) | 1.24(1.04-1.48) | 29.17% | 1.57(1.32-1.87) | 2.16(1.80-2.56) | 1.79(1.24,2.34) |
| India | Both | 266.09(232.61-305.78) | 655.71(543.91-784.43) | 146.42% | 5.9(5.14-6.78) | 5.81(4.84-6.94) | -0.22(-0.32,-0.11) |
| Indonesia | Both | 17.1705(14.37-19.89) | 42.03(31.15-56.90) | 144.78% | 1.82(1.52-2.09) | 2.15(1.59-2.90) | 0.57(0.52,0.61) |
| Iran (Islamic Republic of) | Both | 1.71(1.40-1.99) | 4.86(4.46-5.34) | 184.21% | 0.71(0.57-0.83) | 0.71(0.64-0.78) | -0.06(-0.14,0.02) |
| Iraq | Both | 0.83(0.67-1.01) | 2.15(1.64-2.73) | 159.04% | 1.05(0.84-1.28) | 0.94(0.73-1.15) | -0.57(-0.63,0.51) |
| Israel | Both | 0.39(0.36-0.42) | 1.05(0.94-1.14) | 169.23% | 0.84(0.78-0.90) | 0.89(0.80-0.97) | -0.07(-0.30,0.15) |
| Japan | Both | 17.71(16.86-18.18) | 46.77(38.85-51.15) | 164.09% | 1.08(1.03-1.11) | 1.22(1.07-1.30) | 0.26(0.04,0.57) |
| Jordan | Both | 0.18(0.15-0.21) | 0.63(0.52-0.78) | 250.00% | 1.33(1.09-1.58) | 1.07(0.89-1.30) | -0.89(-0.99,0.79) |
| Kazakhstan | Both | 3.33(3.04-3.98) | 3.93(3.37-4.58) | 18.02% | 2.57(2.35-3.07) | 2.26(1.94-2.62) | -0.77(-0.98,-0.55) |
| Kuwait | Both | 0.07(0.06-0.08) | 0.17(0.14-0.21) | 142.86% | 1.14(1.01-1.26) | 0.72(0.60-0.87) | -1.03(-1.36,0.70) |
| Kyrgyzstan | Both | 0.68(0.60-0.77) | 0.73(0.63-0.86) | 7.35% | 2.19(1.96-2.48) | 1.58(1.37-1.85) | -0.79(-1.00,-0.58) |
| Lao People's Democratic Republic | Both | 0.56(0.38-0.77) | 0.94(0.70-1.21) | 67.86% | 2.74(1.89-3.71) | 2.29(1.74-2.88) | -0.83(-0.94,-0.72) |
| Lebanon | Both | 0.33(0.25-0.41) | 0.68(0.54-0.89) | 106.06% | 1.48(1.15-1.80) | 1.31(1.04-1.72) | -0.08(-0.28,0.11) |
| Malaysia | Both | 2.67(2.34-3.05) | 6.70(5.23-8.52) | 150.94% | 2.97(2.57-3.42) | 2.64(2.08-3.36) | -0.92(-1.18,-0.65) |
| Maldives | Both | 0.02(0.02-0.03) | 0.07(0.06-0.09) | 250.00% | 3.97(3.08-4.71) | 2.90(2.35-3.48) | -1.57(-1.72,-1.42) |
| Mongolia | Both | 0.50(0.41-0.60) | 0.53(0.40-0.70) | 6.00% | 4.80(4.01-5.74) | 2.30(1.79-2.94) | -3.63(-4.26,-3.19) |
| Myanmar | Both | 5.52(4.00-7.43) | 9.60(7.37-12.72) | 73.91% | 2.42(1.79-3.22) | 2.18(1.69-2.86) | -0.44(-0.48,-0.40) |
| Nepal | Both | 4.71(3.28-6.28) | 9.89(7.63-12.17) | 109.98% | 4.91(3.37-6.50) | 4.60(3.57-5.62) | -0.22(-0.49,-0.06) |
| Pakistan | Both | 71.41(60.91-84.06) | 175.66(140.62-221.68) | 145.99% | 12.18(10.34-14.32) | 14.72(11.9-18.34) | 0.51(0.29,0.73) |
| Palestine | Both | 0.07(0.05-0.10) | 0.17(0.14-0.20) | 142.86% | 0.85(0.61-1.14) | 0.76(0.64-0.89) | -0.48(-0.62,0.33) |
| Philippines | Both | 8.05(7.10-9.04) | 15.49(12.68-18.71) | 92.42% | 2.80(2.48-3.12) | 2.10(1.74-2.50) | -1.38(-1.59,1.19) |
| Qatar | Both | 0.01(0.01-0.02) | 0.09(0.06-0.12) | 800.00% | 1.47(1.11-1.87) | 1.27(0.99-1.61) | -0.26(-0.48,0.04) |
| Republic of Korea | Both | 2.71(2.56-2.89) | 7.96(7.09-8.87) | 193.73% | 0.95(0.89-1.03) | 0.92(0.81-1.02) | -0.86(-1.42,-0.29) |
| Saudi Arabia | Both | 0.64(0.46-0.84) | 2.05(1.54-2.68) | 220.31% | 1.10(0.79-1.42) | 1.08(0.85-1.35) | -0.22(-0.33,0.11) |
| Singapore | Both | 0.38(0.36-0.41) | 0.67(0.60-0.75) | 76.32% | 1.80(1.68-1.92) | 0.88(0.79-0.98) | -2.35(-2.51,-2.20) |
| Sri Lanka | Both | 4.14(3.72-4.62) | 10.25(7.40-13.57) | 147.58% | 4.24(3.80-4.71) | 4.21(3.08-5.51) | -0.12(-0.4,0.16) |
| Taiwan China | Both | 4.22(4.05-4.40) | 22.38(17.26-29.94) | 430.33% | 2.52(2.42-2.63) | 5.86(4.50-7.84) | 3.18(2.58,3.79) |
| Tajikistan | Both | 0.26(0.19-0.36) | 0.45(0.36-0.56) | 73.08% | 0.94(0.68-1.33) | 1.04(0.85-1.27) | 0.40(0.21,0.60) |
| Thailand | Both | 12.82(11.20-14.59) | 29.57(22.13-38.21) | 130.66% | 3.90(3.40-4.43) | 2.96(2.23-3.81) | -1.49(-1.71,-1.27) |
| Timor-Leste | Both | 0.04(0.03-0.06) | 0.15(0.11-0.19) | 275.00% | 1.75(1.32-2.24) | 2.02(1.51-2.55) | 0.59(0.36,0.82) |
| Turkmenistan | Both | 0.42(0.39-0.45) | 0.88(0.70-1.11) | 109.52% | 2.14(2.00-2.30) | 2.17(1.73-2.72) | -0.10(-0.34,-0.15) |
| United Arab Emirates | Both | 0.10(0.06-0.15) | 0.75(0.43-1.32) | 650.00% | 1.91(1.04-2.81) | 1.50(0.94-2.44) | -1.00(-1.3,0.72) |
| Uzbekistan | Both | 1.48(1.22-2.05) | 4.59(3.83-5.49) | 210.14% | 1.24(1.01-1.77) | 2.15(1.84-2.51) | 1.80(1.41,2.17) |
| Viet Nam | Both | 12.21(9.72-14.98) | 34.49(26.56-43.39) | 182.47% | 3.06(2.45-3.73) | 3.67(2.87-4.61) | 0.62(0.58,0.65) |
| Afghanistan | Female | 0.27(0.19-0.43) | 0.57(0.36-0.95) | 111.11% | 0.84(0.61-1.18) | 0.85(0.58-1.28) | 0.04(-0.02,0.09) |
| Armenia | Female | 0.10(0.09-0.11) | 0.12(0.10-0.15) | 20.00% | 3.73(2.68-4.87) | 0.52(0.43-0.62) | -2.02(-3.27,-0.75) |
| Azerbaijan | Female | 0.15(0.13-0.18) | 0.33(0.26-0.43) | 120.00% | 1.49(1.18-1.86) | 0.71(0.55-0.93) | 0.73(-0.11,1.59) |
| Bahrain | Female | 0.01(0.01-0.01) | 0.02(0.02-0.03) | 100.00% | 1.32(1.11-1.54) | 0.75(0.61-0.92) | -2.38(-2.66,2.14) |
| Bangladesh | Female | 7.89(5.96-9.76) | 19.57(13.09-26.32) | 148.04% | 0.63(0.50-0.76) | 3.22(2.13-4.32) | 0.50(-0.85,1.86) |
| Bhutan | Female | 0.05(0.03-0.06) | 0.10(0.07-0.13) | 100.00% | 3.86(3.09-4.69) | 3.76(2.82-4.74) | -0.18(-0.28,-0.07) |
| Brunei Darussalam | Female | 0.01(0.01-0.01) | 0.04(0.03-0.04) | 300.00% | 3.74(3.01-4.54) | 2.78(2.38-3.24) | -0.15(-0.44,0.13) |
| Cambodia | Female | 0.41(0.31-0.54) | 1.19(0.86-1.62) | 190.24% | 10.56(8.41-13.1) | 1.88(1.38-2.54) | -0.89(-2.25,0.50) |
| China | Female | 28.33(23.55-33.31) | 50.34(40.59-61.17) | 77.69% | 0.74(0.51-0.94) | 0.50(0.41-0.61) | -1.23(-1.32,-1.14) |
| Cyprus | Female | 0.03(0.03-0.04) | 0.08(0.07-0.09) | 166.67% | 0.88(0.73-1.06) | 0.82(0.70-0.96) | -0.13(-0.28,0.02) |
| Democratic People's Republic of Korea | Female | 0.78(0.61-0.99) | 1.41(1.11-1.78) | 80.77% | 3.59(2.69-4.64) | 0.76(0.59-0.96) | -1.25(-2.34,-0.14) |
| Georgia | Female | 0.22(0.18-0.27) | 0.24(0.20-0.28) | 9.09% | 0.69(0.63-0.72) | 0.65(0.54-0.77) | 0.52(0.18,0.86) |
| India | Female | 80.84(65.37-97.32) | 239.42(191.53-295.44) | 196.17% | 1.20(1.07-1.40) | 4.27(3.41-5.25) | 0.86(-0.06,1.81) |
| Indonesia | Female | 6.93(5.35-9.33) | 15.92(10.82-24.77) | 129.73% | 0.91(0.68-1.40) | 1.62(1.11-2.45) | 0.53(0.11,0.95) |
| Iran (Islamic Republic of) | Female | 0.57(0.47-0.64) | 2.08(1.88-2.29) | 264.91% | 0.50(0.41-0.58) | 0.61(0.55-0.68) | 0.72(0.60,0.84) |
| Iraq | Female | 0.32(0.24-0.40) | 0.97(0.72-1.26) | 203.13% | 0.78(0.59-0.99) | 0.81(0.62-1.01) | 0.07(0.03,0.11) |
| Israel | Female | 0.17(0.15-0.18) | 0.43(0.36-0.49) | 152.94% | 0.67(0.61-0.74) | 0.62(0.54-0.71) | -0.57(-0.77,0.36) |
| Japan | Female | 6.38(5.86-6.64) | 21.60(16.30-24.92) | 238.56% | 0.83(0.65-1.03) | 0.84(0.69-0.94) | 0.41(0.14,0.68) |
| Jordan | Female | 0.06(0.05-0.07) | 0.20(0.15-0.25) | 233.33% | 0.93(0.74-1.14) | 0.75(0.60-0.93) | -1.08(-1.31,0.86) |
| Kazakhstan | Female | 0.92(0.82-1.08) | 1.31(1.10-1.57) | 42.39% | 0.55(0.50-0.61) | 1.32(1.11-1.57) | 0.74(0.03,1.47) |
| Kuwait | Female | 0.02(0.02-0.03) | 0.06(0.05-0.08) | 200.00% | 0.96(0.82-1.10) | 0.62(0.48-0.80) | -0.54(-1.06,0.01) |
| Kyrgyzstan | Female | 0.18(0.16-0.20) | 0.24(0.20-0.28) | 33.33% | 1.17(1.07-1.28) | 0.93(0.79-1.12) | 0.01(-0.28,0.29) |
| Lao People's Democratic Republic | Female | 0.18(0.12-0.27) | 0.33(0.24-0.46) | 83.33% | 1.73(1.18-2.48) | 1.60(1.19-2.21) | -0.52(-0.62,-0.42) |
| Lebanon | Female | 0.10(0.09-0.13) | 0.27(0.21-0.36) | 170.00% | 0.95(0.79-1.16) | 0.95(0.74-1.28) | 0.06(0.03,0.09) |
| Malaysia | Female | 1.18(1.02-1.35) | 3.03(2.36-3.84) | 156.78% | 3.75(2.81-4.72) | 2.48(1.95-3.12) | -0.78(-1.09,-0.46) |
| Maldives | Female | 0.01(0.01-0.02) | 0.04(0.03-0.05) | 300.00% | 0.69(0.57-0.80) | 3.68(2.92-4.52) | -0.24(-1.82,1.37) |
| Mongolia | Female | 0.22(0.18-0.27) | 0.16(0.13-0.21) | -27.27% | 2.00(1.61-2.43) | 1.36(1.05-1.77) | -4.46(-5.34,-3.57) |
| Myanmar | Female | 2.04(1.45-3.07) | 3.83(2.86-5.60) | 87.75% | 0.96(0.85-1.08) | 1.59(1.20-2.34) | -0.09(-0.60,0.42) |
| Nepal | Female | 1.64(1.27-2.08) | 4.17(3.09-5.26) | 154.27% | 1.74(1.27-2.51) | 3.74(2.78-4.71) | 0.56(0.02,1.11) |
| Pakistan | Female | 28.04(22.65-34.64) | 76.11(58.29-99.04) | 171.43% | 2.72(2.33-3.14) | 13.37(10.43-17.04) | 1.62(0.51,2.75) |
| Palestine | Female | 0.03(0.02-0.04) | 0.07(0.06-0.09) | 133.33% | 0.61(0.45-0.79) | 0.63(0.52-0.77) | 0.04(-0.11,0.20) |
| Philippines | Female | 3.23(2.80-3.69) | 6.07(4.79-7.62) | 87.93% | 1.24(1.06-1.43) | 1.61(1.28-2.00) | -1.32(-1.87,-0.77) |
| Qatar | Female | 0.00(0.00-0.01) | 0.03(0.02-0.03) | #VALUE! | 1.45(0.98-1.95) | 1.73(1.37-2.16) | 1.09(0.82,1.35) |
| Republic of Korea | Female | 0.86(0.78-0.95) | 2.94(2.48-3.40) | 241.86% | 1.09(0.99-1.21) | 0.59(0.51-0.68) | -0.87(-1.43,-0.29) |
| Saudi Arabia | Female | 0.25(0.18-0.33) | 0.87(0.66-1.15) | 248.00% | 1.01(0.72-1.35) | 1.17(0.91-1.49) | 0.36(0.23,0.48) |
| Singapore | Female | 0.13(0.12-0.14) | 0.25(0.21-0.29) | 92.31% | 0.87(0.80-0.93) | 0.62(0.52-0.72) | -1.91(-2.18,-1.66) |
| Sri Lanka | Female | 1.45(1.25-1.68) | 3.36(2.54-4.46) | 131.72% | 4.99(3.79-6.65) | 2.50(1.90-3.30) | -1.64(-1.99,-1.28) |
| Taiwan China | Female | 0.62(0.58-0.66) | 2.36(1.80-3.05) | 280.65% | 1.69(1.31-2.17) | 1.13(0.86-1.46) | 0.65(0.13,1.17) |
| Tajikistan | Female | 0.12(0.08-0.15) | 0.20(0.16-0.25) | 66.67% | 0.67(0.58-0.77) | 0.85(0.68-1.06) | 0.56(0.42,0.69) |
| Thailand | Female | 5.07(4.3-5.93) | 13.24(9.85-17.04) | 161.14% | 1.47(1.16-1.91) | 2.39(1.78-3.07) | -0.92(-1.64,-0.18) |
| Timor-Leste | Female | 0.02(0.01-0.02) | 0.06(0.05-0.08) | 200.00% | 3.03(2.59-3.52) | 1.62(1.25-2.09) | -0.18(-0.76,0.40) |
| Turkmenistan | Female | 0.12(0.11-0.13) | 0.30(0.24-0.38) | 150.00% | 2.67(2.22-3.10) | 1.37(1.09-1.72) | 0.52(-0.26,1.32) |
| United Arab Emirates | Female | 0.02(0.01-0.03) | 0.10(0.05-0.19) | 400.00% | 1.03(0.54-1.90) | 0.93(0.52-1.77) | -0.17(-0.85,0.51) |
| Uzbekistan | Female | 0.61(0.47-0.93) | 1.74(1.42-2.10) | 185.25% | 2.37(2.06-2.66) | 1.54(1.28-1.81) | 1.14(0.47,1.82) |
| Viet Nam | Female | 4.61(3.68-5.61) | 9.48(7.35-12.10) | 105.64% | 3.07(2.56-3.62) | 1.86(1.44-2.37) | -0.70(-1.02,-0.37) |
| Afghanistan | Male | 0.61(0.30-1.08) | 0.83(0.46-1.37) | 36.07% | 1.68(0.87-2.92) | 1.34(0.81-2.14) | -0.80(-0.83,0.76) |
| Armenia | Male | 0.26(0.23-0.31) | 0.35(0.29-0.42) | 34.62% | 2.18(1.89-2.51) | 1.95(1.61-2.33) | -0.49(-0.80,-0.18) |
| Azerbaijan | Male | 0.23(0.18-0.28) | 0.60(0.43-0.81) | 160.87% | 1.09(0.88-1.39) | 1.52(1.12-2.05) | 1.8(1.33,2.27) |
| Bahrain | Male | 0.02(0.02-0.03) | 0.08(0.05-0.10) | 300.00% | 2.59(2.06-3.18) | 1.43(1.07-1.84) | -2.44(-2.81,2.11) |
| Bangladesh | Male | 22.43(14.92-30.68) | 32.29(22.14-49.72) | 43.96% | 8.51(5.71-11.56) | 4.84(3.35-7.34) | -2.06(-2.22,-1.88) |
| Bhutan | Male | 0.09(0.06-0.13) | 0.17(0.11-0.24) | 88.89% | 7.26(4.30-10.24) | 5.85(3.94-8.24) | -0.81(-0.97,-0.65) |
| Brunei Darussalam | Male | 0.03(0.02-0.04) | 0.06(0.05-0.07) | 100.00% | 7.49(5.70-9.15) | 5.40(4.42-6.48) | -0.71(-1.08,-0.34) |
| Cambodia | Male | 0.70(0.47-1.04) | 2.01(1.44-2.68) | 187.14% | 3.59(2.44-5.30) | 4.19(3.01-5.56) | 0.55(0.45,0.65) |
| China | Male | 45.71(37.24-54.47) | 176.08(140.94-218.97) | 285.21% | 1.23(1.02-1.44) | 1.95(1.59-2.40) | 2.55(2.16,2.95) |
| Cyprus | Male | 0.06(0.05-0.07) | 0.12(0.10-0.14) | 100.00% | 1.67(1.40-1.98) | 1.38(1.15-1.66) | -0.71(-0.86,0.56) |
| Democratic People's Republic of Korea | Male | 1.21(0.82-1.73) | 2.49(1.80-3.45) | 105.79% | 1.85(1.32-2.54) | 1.87(1.42-2.54) | 0.18(0.10,0.26) |
| Georgia | Male | 0.74(0.61-0.92) | 1.01(0.84-1.20) | 36.49% | 2.94(2.45-3.65) | 4.17(3.46-4.95) | 2.00(1.34,2.66) |
| India | Male | 185.25(149.75-224.40) | 416.29(321.02-527.75) | 124.72% | 7.81(6.19-9.48) | 7.40(5.75-9.35) | -0.30(-0.40,-0.21) |
| Indonesia | Male | 10.24(8.29-12.17) | 26.12(18.99-35.88) | 155.08% | 2.20(1.78-2.59) | 2.73(1.98-3.71) | 0.82(0.77,0.88) |
| Iran (Islamic Republic of) | Male | 1.14(0.91-1.38) | 2.78(2.51-3.12) | 143.86% | 0.92(0.72-1.12) | 0.80(0.72-0.89) | -0.54(-0.61,0.47) |
| Iraq | Male | 0.52(0.39-0.66) | 1.18(0.89-1.53) | 126.92% | 1.34(1.01-1.71) | 1.09(0.83-1.37) | -0.97(-1.05,0.88) |
| Israel | Male | 0.22(0.20-0.25) | 0.62(0.56-0.69) | 181.82% | 1.04(0.94-1.14) | 1.19(1.07-1.32) | 0.22(-0.06,0.50) |
| Japan | Male | 11.33(10.99-11.58) | 25.18(22.54-26.67) | 122.24% | 1.58(1.52-1.61) | 1.65(1.50-1.73) | -0.01(-0.31,0.30) |
| Jordan | Male | 0.12(0.09-0.15) | 0.43(0.32-0.57) | 258.33% | 1.72(1.35-2.13) | 1.35(1.02-1.78) | -0.83(-1.00,0.68) |
| Kazakhstan | Male | 2.41(2.17-2.97) | 2.63(2.20-3.10) | 9.13% | 4.79(4.32-5.89) | 3.69(3.12-4.33) | -1.27(-1.49,-1.06) |
| Kuwait | Male | 0.05(0.04-0.05) | 0.11(0.09-0.14) | 120.00% | 1.24(1.07-1.41) | 0.78(0.61-0.98) | -1.23(-1.55,0.92) |
| Kyrgyzstan | Male | 0.51(0.44-0.59) | 0.50(0.42-0.59) | -1.96% | 3.99(3.48-4.62) | 2.44(2.07-2.90) | -1.29(-1.54,-1.05) |
| Lao People's Democratic Republic | Male | 0.39(0.23-0.60) | 0.62(0.44-0.80) | 58.97% | 3.87(2.4-5.93) | 3.03(2.14-3.84) | -1.02(-1.15,-0.90) |
| Lebanon | Male | 0.23(0.16-0.29) | 0.41(0.31-0.55) | 78.26% | 2.02(1.44-2.58) | 1.76(1.33-2.38) | -0.02(-0.33,0.28) |
| Malaysia | Male | 1.49(1.22-1.81) | 3.68(2.71-4.85) | 146.98% | 3.30(2.69-3.97) | 2.80(2.10-3.66) | -1.23(-1.65,-0.89) |
| Maldives | Male | 0.02(0.01-0.02) | 0.03(0.03-0.04) | 50.00% | 3.46(2.40-4.83) | 2.21(1.75-2.74) | -2.12(-2.28,-1.94) |
| Mongolia | Male | 0.28(0.22-0.36) | 0.37(0.28-0.50) | 32.14% | 6.12(4.84-7.50) | 3.59(2.77-4.57) | -2.90(-3.33,-2.45) |
| Myanmar | Male | 3.48(2.27-5.39) | 5.77(4.21-7.52) | 65.80% | 3.18(2.13-4.83) | 2.93(2.14-3.75) | -0.30(-0.35,-0.24) |
| Nepal | Male | 3.07(1.89-4.44) | 5.73(4.30-7.30) | 86.64% | 6.17(3.80-8.76) | 5.53(4.17-7.04) | -0.34(-0.68,0.01) |
| Pakistan | Male | 43.37(36.34-52.64) | 99.55(70.44-136.28) | 129.54% | 13.53(11.24-16.40) | 15.97(11.47-21.55) | 0.40(0.13,0.66) |
| Palestine | Male | 0.04(0.03-0.06) | 0.09(0.08-0.11) | 125.00% | 1.16(0.79-1.66) | 0.94(0.79-1.12) | -0.87(-1.00,0.73) |
| Philippines | Male | 4.83(4.12-5.76) | 9.42(7.10-12.29) | 95.03% | 3.25(2.78-3.90) | 2.64(2.02-3.41) | -1.07(-1.27,-0.87) |
| Qatar | Male | 0.01(0.01-0.01) | 0.06(0.04-0.09) | 500.00% | 1.50(1.09-1.97) | 1.11(0.82-1.48) | -0.96(-1.25,0.67) |
| Republic of Korea | Male | 1.86(1.73-2.02) | 5.03(4.33-5.74) | 170.43% | 1.57(1.45-1.72) | 1.32(1.13-1.49) | -1.40(-2.01,-0.78) |
| Saudi Arabia | Male | 0.40(0.27-0.52) | 1.18(0.85-1.60) | 195.00% | 1.16(0.80-1.51) | 1.01(0.76-1.30) | -0.65(-0.77,0.53) |
| Singapore | Male | 0.26(0.24-0.28) | 0.42(0.37-0.48) | 61.54% | 2.63(2.42-2.86) | 1.17(1.03-1.33) | -2.59(-2.77,-2.40) |
| Sri Lanka | Male | 2.70(2.34-3.08) | 6.90(4.78-9.50) | 155.56% | 5.45(4.75-6.20) | 6.42(4.53-8.77) | 0.79(0.40,1.20) |
| Taiwan China | Male | 3.60(3.44-3.77) | 20.03(15.36-26.61) | 456.39% | 4.05(3.87-4.24) | 10.95(8.36-14.52) | 3.73(3.05,4.40) |
| Tajikistan | Male | 0.15(0.11-0.24) | 0.25(0.20-0.32) | 66.67% | 1.22(0.87-2.08) | 1.27(1.02-1.59) | 0.28(-0.03,0.59) |
| Thailand | Male | 7.75(6.43-9.24) | 16.34(11.89-21.63) | 110.84% | 4.75(3.96-5.66) | 3.53(2.58-4.62) | -1.58(-1.80,-1.35) |
| Timor-Leste | Male | 0.03(0.02-0.04) | 0.09(0.06-0.13) | 200.00% | 1.99(1.22-2.83) | 2.42(1.55-3.29) | 0.85(0.55,1.16) |
| Turkmenistan | Male | 0.30(0.28-0.33) | 0.58(0.46-0.73) | 93.33% | 3.62(3.32-3.97) | 3.20(2.52-4.01) | -0.79(-1.05,-0.52) |
| United Arab Emirates | Male | 0.08(0.05-0.12) | 0.65(0.37-1.13) | 712.50% | 2.46(1.29-3.60) | 1.72(1.07-2.72) | -1.56(-1.82,1.31) |
| Uzbekistan | Male | 0.87(0.72-1.19) | 2.86(2.35-3.43) | 228.74% | 1.68(1.40-2.42) | 2.94(2.46-3.47) | 1.78(1.35,2.20) |
| Viet Nam | Male | 7.61(5.94-9.77) | 25.02(18.78-32.14) | 228.78% | 4.46(3.49-5.62) | 6.03(4.65-7.69) | 1.11(1.07,1.14) |
