# Supplementary material for: Burden of oral cancer in Asia from 1990 to 2019: Estimates from the Global Burden of Disease 2019 study
Source: PLoS One. 2022 Mar 24;17(3):e0265950. doi: 10.1371/journal.pone.0265950 (PMC8947401; doi:10.1371/journal.pone.0265950)
Supplement: S4 Table — (DOC) [file pone.0265950.s004.doc]

**Supplementary Table 4. The DALY and age-standardized DALY rate of Oral cancer in 1990 and 2019, and its temporal trends from 1990 to 2019.**

| Nation | sex | DALY No.*10^2^  (95%UI) | | Change in absolute number(%) | age-standardized DALY rate per 100,000 No.(95%UI) | | 1990-2019 EAPC No.(95%CI) |
| --- | --- | --- | --- | --- | --- | --- | --- |
|  |  | 1990 | 2019 |  | 1990 | 2019 |  |
| Over all  Asia | Both | 17230.76(15680.80-19013.21) | 39548.26(35003.48-44708.67) | 129.52% | 74.33(67.43-81.94) | 79.03(70.13-89.15) | 0.20(0.16,0.24) |
| East Asia | Both | 2433.22(2129.35,2742.17) | 6563.36(5590.63,7748.15) | 169.74% | 24.81(21.77,27.83) | 31.1(26.57,36.49) | 1.37(1.11,1.62) |
| Southeast Asia | Both | 1881.13(1694.14,2040.10) | 4064.3(3373.12,4852.97) | 116.06% | 65.46(58.88,70.81) | 62.87(52.51,75.04) | -0.12(-0.15,-0.08) |
| Central Asia | Both | 252.41(234.97,283.43) | 416.28(375.67,464.78) | 64.92% | 49.25(45.88,55.54) | 49.31(44.63,54.77) | -0.29(-0.66,0.08) |
| High-income Asia Pacific | Both | 527.50(513.32,538.74) | 968.03(877.63,1028.05) | 83.51% | 25.95(25.17,26.51) | 25.7(24.03,27.05) | -0.52(-0.56,-0.47) |
| South Asia | Both | 11786.31(10435.70,13354.23) | 26898.83(23168.66,31458.19) | 128.22% | 175.07(152.58,198.85) | 173.17(149.26,202.43) | -0.15(-0.23,-0.07) |
| West Asia | Both | 454.27(382.49,527.09) | 1003.73(867.62,1170.99) | 120.95% | 23.17(19.38,26.89) | 20.62(17.95,23.91) | -0.33(-0.40,-0.25) |
| Armenia | Both | 10.95(9.81-12.37) | 12.01(9.89-14.41) | 9.68% | 35.82(32.22-40.09) | 29.29(24.16-34.89) | -0.79(-1.10,-0.47) |
| Afghanistan | Both | 25.81(14.28-39.74) | 47.11(27.84-66.56) | 82.53% | 33.62(18.93-51.46) | 27.97(16.90-38.37) | -0.70(-0.74,-0.66) |
| Azerbaijan | Both | 11.52(9.93-13.49) | 27.45(21.43-34.42) | 138.28% | 20.67(17.89-24.29) | 26.32(20.67-32.88) | 1.25(0.84,1.65) |
| Bahrain | Both | 1.04(0.87-1.24) | 3.11(2.27-4.10) | 199.04% | 46.75(38.66-55.39) | 25.01(18.95-31.99) | -2.66(-2.91,-2.43) |
| Bangladesh | Both | 935.43(682.5-1213.19) | 1446.51(986.87-2047.83) | 54.64% | 171.71(124.61-224.07) | 103.95(71.30-146.15) | -1.80(-1.93,-1.68) |
| Bhutan | Both | 4.50(2.95-6.08) | 7.38(5.05-10.47) | 64.00% | 147.44(98.68-197.98) | 120.34(83.62-166.13) | -0.82(-0.96,-0.68) |
| Brunei Darussalam | Both | 1.34(1.14-1.56) | 2.82(2.43-3.29) | 110.45% | 110.64(92.45-129.03) | 82.53(72.08-95.00) | -0.86(-1.09,-0.62) |
| Cambodia | Both | 33.39(24.43-43.91) | 87.50(64.55-114.43) | 162.05% | 64.51(47.86-85.17) | 68.06(50.60-88.47) | 0.15(0.06,0.23) |
| China | Both | 2226.47(1925.34-2524.03) | 5758.05(4795.22-6907.43) | 158.62% | 23.63(20.48-26.66) | 28.27(23.59-33.71) | 1.24(0.95,1.52) |
| Cyprus | Both | 2.40(2.08-2.71) | 4.70(4.04-5.39) | 95.83% | 30.16(26.18-34.16) | 25.74(22.16-29.56) | -0.55(-0.64,-0.47) |
| Democratic People's Republic of Korea | Both | 63.95(46.36-87.16) | 109.43(82.22-147.43) | 71.12% | 34.45(25.58-46.06) | 33.26(25.21-44.74) | -0.06(-0.11,0.00) |
| Georgia | Both | 28.08(23.48-33.95) | 32.70(27.06-39.12) | 16.45% | 44.61(37.52-53.69) | 60.59(50.17-72.51) | 1.68(1.14,2.23) |
| India | Both | 8476.15(7453.09-9619.13) | 19226.64(15853.85-23180.25) | 126.83% | 156.3(137.01-178.41) | 154.90(128.07-186.18) | -0.13(-0.22,-0.05) |
| Indonesia | Both | 518.01(435.42-609.11) | 1131.43(833.76-1533.09) | 118.42% | 45.54(38.08-52.90) | 48.92(36.22-66.05) | 0.24(0.19,0.29) |
| Iran(Islamic Republic of) | Both | 50.85(42.64-58.63) | 124.33(115.07-135.23) | 144.50% | 16.85(13.89-19.53) | 15.98(14.70-17.44) | -0.23(-0.31,-0.14) |
| Iraq | Both | 25.47(20.36-31.31) | 66.50(49.51-87.04) | 161.09% | 28.21(22.57-34.63) | 23.98(18.05-30.78) | -0.75(-0.82,-0.69) |
| Israel | Both | 9.19(8.60-9.85) | 22.07(20.06-24.12) | 140.15% | 19.61(18.29-20.96) | 20.4(18.55-22.30) | -0.18(-0.42,0.07) |
| Japan | Both | 435.67(421.91-444.97) | 770.39(683.75-819.94) | 76.83% | 26.15(25.32-26.71) | 27.73(25.76-29.06) | -0.01(-0.36,0.33) |
| Jordan | Both | 5.52(4.57-6.62) | 18.59(15.21-22.83) | 236.78% | 33.14(27.42-39.63) | 24.07(19.79-29.48) | -1.36(-1.48,-1.23) |
| Kazakhstan | Both | 98.35(89.55-118.27) | 111.02(95.00-130.72) | 12.88% | 71.35(65.18-85.92) | 59.09(50.77-69.23) | -1.03(-1.27,-0.80) |
| Kuwait | Both | 2.29(2.05-2.54) | 4.86(3.98-5.89) | 112.23% | 27.50(24.55-30.45) | 15.30(12.65-18.51) | -1.48(-1.82,-1.13) |
| Kyrgyzstan | Both | 20.54(18.16-23.31) | 21.19(18.07-24.95) | 3.16% | 63.63(56.4-71.97) | 40.82(35.02-47.92) | -1.24(-1.45,-1.02) |
| Lao People's Democratic Republic | Both | 17.30(11.38-23.93) | 27.04(20.11-35.42) | 56.30% | 72.91(48.86-100.33) | 55.11(41.09-71.84) | -1.16(-1.29,-1.04) |
| Lebanon | Both | 9.61(7.20-12.09) | 17.19(13.09-22.78) | 78.88% | 38.69(29.29-48.31) | 33.00(25.02-43.86) | -0.25(-0.49,-0.02) |
| Malaysia | Both | 76.41(66.98-86.77) | 178.80(136.52-229.72) | 134.00% | 73.77(64.71-84.17) | 63.01(48.53-80.89) | -1.00(-1.33,-0.68) |
| Maldives | Both | 0.83(0.60-1.03) | 1.73(1.41-2.09) | 108.43% | 86.69(65.53-103.31) | 54.72(44.52-65.96) | -2.12(-2.28,-1.94) |
| Mongolia | Both | 14.20(11.63-17.12) | 16.49(12.38-21.90) | 16.13% | 122.69(101.00-148.47) | 60.04(46.23-78.96) | -3.61(-4.12,-3.10) |
| Myanmar | Both | 167.64(120.04-226.14) | 259.68(194.13-348.24) | 54.90% | 63.91(46.23-86.22) | 52.59(39.81-69.72) | -0.77(-0.82,-0.73) |
| Nepal | Both | 150.23(106.35-199.60) | 277.41(210.46-350.56) | 84.66% | 133.35(93.33-177.57) | 115.51(88.39-144.26) | -0.47(-0.78,-0.17) |
| Pakistan | Both | 2220.00(1884.51-2624.36) | 5940.89(4713.17-7548.97) | 167.61% | 343.14(293.41-405.54) | 421.87(338.19-535.02) | 0.56(0.32,0.81) |
| Palestine | Both | 2.01(1.43-2.76) | 4.84(4.03-5.72) | 140.80% | 20.63(14.69-28.03) | 17.63(14.71-20.68) | -0.65(-0.81,-0.48) |
| Philippines | Both | 242.38(212.69-269.56) | 434.44(353.93-527.79) | 79.24% | 69.30(60.80-77.68) | 50.83(41.65-61.35) | -1.50(-1.71,-1.28) |
| Qatar | Both | 0.49(0.37-0.61) | 2.97(2.12-4.02) | 506.12% | 32.35(24.95-41.34) | 24.52(18.82-31.68) | -0.83(-1.06,-0.60) |
| Republic of Korea | Both | 79.55(74.96-85.18) | 178.94(160.78-200.24) | 124.94% | 23.42(22.12-25.03) | 20.70(18.68-23.08) | -1.03(-1.55,-0.52) |
| Saudi Arabia | Both | 19.76(14.21-26.20) | 71.67(52.8-96.87) | 262.70% | 27.17(19.39-35.60) | 27.13(20.77-34.89) | -0.12(-0.22,-0.03) |
| Singapore | Both | 10.94(10.23-11.67) | 15.87(14.34-17.62) | 45.06% | 44.07(41.22-46.89) | 19.93(18.08-22.04) | -2.66(-2.83,-2.49) |
| Sri Lanka | Both | 107.69(96.30-120.11) | 244.89(175.24-328.03) | 127.40% | 94.36(84.68-105.32) | 94.64(68.19-125.68) | 0.00(-0.25,0.26) |
| Taiwan China | Both | 142.80(136.33-149.12) | 695.87(529.72-936.21) | 387.30% | 79.03(75.52-82.48) | 188.97(143.03-254.04) | 3.22(2.55,3.90) |
| Tajikistan | Both | 7.14(5.88-8.99) | 13.66(10.82-17.17) | 91.32% | 23.73(19.00-30.95) | 23.90(19.23-29.77) | -0.07(-0.32,0.17) |
| Thailand | Both | 359.54(314.29-409.21) | 677.76(490.89-893.14) | 88.51% | 91.00(79.63-103.66) | 67.00(48.81-87.68) | -1.66(-1.89,-1.43) |
| Timor-Leste | Both | 1.54(1.14-2.04) | 4.05(2.74-5.35) | 162.99% | 43.25(32.13-56.39) | 47.11(33.32-61.6) | 0.37(0.08,0.66) |
| Turkmenistan | Both | 13.32(12.45-14.34) | 27.81(22.01-35.37) | 108.78% | 60.30(56.36-64.87) | 61.39(48.74-77.86) | -0.06(-0.30,0.19) |
| United Arab Emirates | Both | 3.88(2.47-5.69) | 29.89(16.75-53.06) | 670.36% | 48.15(27.34-70.33) | 37.64(22.94-63.13) | -1.06(-1.27,-0.86) |
| Uzbekistan | Both | 48.31(40.87-62.89) | 153.96(128.1-184.82) | 218.69% | 36.32(30.43-48.37) | 57.88(48.62-68.80) | 1.38(1.03,1.75) |
| Viet Nam | Both | 348.42(274.31-439.01) | 998.11(746.9-1283.94) | 186.47% | 81.81(64.44-103.43) | 96.52(73.31-123.16) | 0.59(0.57,0.61) |
| Afghanistan | Female | 8.40(5.35-14.09) | 19.58(11.84-34.81) | 133.10% | 22.05(14.66-35.63) | 22.12(13.86-36.98) | -0.05(-0.10,-0.01) |
| Armenia | Female | 2.90(2.41-3.46) | 2.75(2.24-3.30) | -5.17% | 17.74(15.00-20.93) | 12.55(10.32-14.97) | -1.22(-1.47,-0.98) |
| Azerbaijan | Female | 4.41(3.77-5.09) | 9.7(7.59-12.25) | 119.95% | 14.15(12.00-16.42) | 17.72(13.93-22.42) | 1.06(0.77,1.34) |
| Bahrain | Female | 0.27(0.22-0.32) | 0.67(0.52-0.85) | 148.15% | 28.72(24.01-34.03) | 15.47(12.35-19.26) | -2.67(-2.99,-2.34) |
| Bangladesh | Female | 246.32(180.23-302.90) | 547.09(366.62-740.90) | 122.11% | 96.05(72.43-117.42) | 79.97(53.63-107.77) | -0.71(-0.83,-0.60) |
| Bhutan | Female | 1.41(0.91-1.84) | 2.66(1.89-3.53) | 88.65% | 95.34(65.09-123.99) | 90.48(65.54-118.73) | -0.39(-0.51,-0.26) |
| Brunei Darussalam | Female | 0.39(0.31-0.48) | 1.05(0.87-1.26) | 169.23% | 62.16(52.60-74.10) | 60.34(51.01-71.09) | -0.10(-0.28,0.08) |
| Cambodia | Female | 11.89(8.68-16.70) | 29.87(21.41-41.77) | 151.22% | 41.12(30.76-56.06) | 42.05(30.27-58.22) | -0.02(-0.07,0.02) |
| China | Female | 840.52(690.25-993.92) | 1210.21(977.02-1472.67) | 43.98% | 17.75(14.66-21.05) | 11.94(9.65-14.51) | -1.57(-1.66,-1.48) |
| Cyprus | Female | 0.78(0.66-0.89) | 1.64(1.37-1.92) | 110.26% | 18.95(16.21-21.74) | 17.12(14.36-19.94) | -0.30(-0.40,-0.20) |
| Democratic People's Republic of Korea | Female | 23.19(17.54-30.44) | 34.96(26.88-45.17) | 50.75% | 22.42(17.24-28.98) | 19.66(14.98-25.67) | -0.46(-0.51,-0.41) |
| Georgia | Female | 5.44(4.33-6.55) | 4.99(4.09-5.95) | -8.27% | 15.53(12.35-18.73) | 15.99(13.14-19.20) | 0.50(0.22,0.77) |
| India | Female | 2569.91(2104.37-3072.76) | 6572.54(5258.01-8149.96) | 155.75% | 99.46(80.51-119.81) | 106.8(85.53-132.12) | 0.02(-0.15,0.18) |
| Indonesia | Female | 202.95(153.98-284.96) | 399.56(270.19-628.42) | 96.88% | 35.12(26.98-48.00) | 34.58(23.60-53.53) | -0.20(-0.31,-0.08) |
| Iran(Islamic Republic of) | Female | 17.41(15.34-19.47) | 52.99(48.98-57.31) | 204.37% | 11.85(9.97-13.41) | 13.71(12.63-14.91) | 0.50(0.39,0.62) |
| Iraq | Female | 9.64(7.43-12.06) | 30.59(22.43-40.66) | 217.32% | 21.00(15.89-26.42) | 21.49(15.94-28.22) | 0.03(-0.01,0.08) |
| Israel | Female | 3.69(3.36-4.05) | 7.68(6.80-8.65) | 108.13% | 14.71(13.45-16.10) | 12.98(11.52-14.56) | -0.72(-0.91,-0.54) |
| Japan | Female | 136.69(130.17-140.88) | 290.29(237.12-325.28) | 112.37% | 15.28(14.59-15.72) | 17.78(15.95-19.46) | 0.35(0.02,0.68) |
| Jordan | Female | 1.79(1.47-2.20) | 5.82(4.46-7.47) | 225.14% | 22.27(18.22-27.34) | 16.23(12.64-20.49) | -1.53(-1.78,-1.27) |
| Kazakhstan | Female | 24.57(21.89-28.69) | 35.43(29.5-43.48) | 44.20% | 31.40(28.01-36.79) | 34.05(28.45-41.72) | 0.19(-0.10,0.49) |
| Kuwait | Female | 0.68(0.60-0.76) | 1.72(1.28-2.33) | 152.94% | 22.28(19.33-25.28) | 12.62(9.61-16.68) | -1.13(-1.65,-0.61) |
| Kyrgyzstan | Female | 4.75(4.19-5.37) | 6.44(5.32-7.85) | 35.58% | 26.03(23.01-29.42) | 22.99(19.12-27.82) | -0.33(-0.52,-0.13) |
| Lao People's Democratic Republic | Female | 5.26(3.35-8.32) | 8.84(6.23-12.80) | 68.06% | 43.16(28.31-65.88) | 36.04(25.75-51.7) | -0.87(-0.98,-0.76) |
| Lebanon | Female | 2.89(2.32-3.62) | 6.39(4.85-8.46) | 121.11% | 23.47(18.99-29.14) | 22.52(17.07-29.87) | -0.13(-0.18,-0.09) |
| Malaysia | Female | 32.06(28.13-35.97) | 75.63(58.04-96.02) | 135.90% | 62.15(54.40-70.08) | 55.01(42.27-69.51) | -0.75(-0.98,-0.52) |
| Maldives | Female | 0.38(0.25-0.62) | 0.88(0.71-1.07) | 131.58% | 99.36(70.71-145.27) | 65.23(52.71-79.38) | -1.91(-2.08,-1.74) |
| Mongolia | Female | 6.09(4.94-7.46) | 4.80(3.62-6.38) | -21.18% | 93.59(76.12-113.45) | 34.27(26.21-45.15) | -4.83(-5.47,-4.18) |
| Myanmar | Female | 59.89(40.21-95.71) | 94.89(68.97-140.58) | 58.44% | 43.69(30.28-67.81) | 35.62(26.15-52.62) | -0.91(-1.03,-0.78) |
| Nepal | Female | 52.40(40.4-66.6) | 117.01(85.19-150.99) | 123.30% | 94.62(73.44-120.29) | 92.17(67.98-117.77) | -0.14(-0.35,0.06) |
| Pakistan | Female | 883.19(716.15-1081.03) | 2565.86(1933.35-3385.98) | 190.52% | 293.08(236.28-360.39) | 375.37(286.27-490.06) | 0.75(0.57,0.93) |
| Palestine | Female | 0.79(0.58-1.02) | 2.06(1.68-2.49) | 160.76% | 14.80(11.01-19.18) | 14.63(11.88-17.59) | -0.10(-0.27,0.08) |
| Philippines | Female | 87.25(76.49-99.54) | 151.73(117.57-193.63) | 73.90% | 51.55(44.90-59.02) | 35.15(27.51-44.28) | -1.73(-1.94,-1.53) |
| Qatar | Female | 0.13(0.11-0.17) | 0.82(0.61-1.09) | 530.77% | 31.47(22.53-41.61) | 33.56(26.43-43.14) | 0.59(0.34,0.84) |
| Republic of Korea | Female | 23.12(21.08-25.62) | 56.5(49.01-63.94) | 144.38% | 12.55(11.46-13.94) | 12.61(10.94-14.18) | -0.50(-0.92,-0.08) |
| Saudi Arabia | Female | 7.46(5.25-10.03) | 30.50(22.55-40.87) | 308.85% | 24.93(17.75-33.20) | 29.27(22.53-37.67) | 0.43(0.31,0.54) |
| Singapore | Female | 3.62(3.31-3.94) | 5.46(4.73-6.35) | 50.83% | 27.25(24.95-29.72) | 13.79(12.02-16.04) | -2.37(-2.54,-2.21) |
| Sri Lanka | Female | 36.77(31.67-42.68) | 71.64(53.58-96.37) | 94.83% | 65.32(56.26-75.66) | 50.93(38.33-68.07) | -1.44(-1.71,-1.17) |
| Taiwan China | Female | 17.77(16.55-19) | 55.49(41.94-72.56) | 212.27% | 21.80(20.32-23.27) | 28.38(21.35-37.11) | 1.13(0.93,1.32) |
| Tajikistan | Female | 3.07(2.50-3.62) | 6.22(4.85-7.95) | 102.61% | 18.44(14.66-21.96) | 20.21(16.01-25.65) | 0.23(0.10,0.36) |
| Thailand | Female | 121.35(103.97-141.12) | 237.06(177.79-307.50) | 95.35% | 61.78(52.85-71.85) | 43.94(32.92-57.36) | -1.76(-2.07,-1.47) |
| Timor-Leste | Female | 0.59(0.43-0.79) | 1.53(1.13-2.01) | 159.32% | 35.14(27.22-44.63) | 35.90(27.00-46.70) | 0.03(-0.18,0.24) |
| Turkmenistan | Female | 3.71(3.39-4.10) | 9.52(7.41-12.34) | 156.60% | 30.29(27.74-33.51) | 40.10(31.46-51.50) | 1.40(1.03,1.78) |
| United Arab Emirates | Female | 0.56(0.34-0.96) | 3.87(1.96-7.60) | 591.07% | 24.94(13.90-46.23) | 22.33(12.25-42.97) | -0.29(-0.77,0.19) |
| Uzbekistan | Female | 19.38(15.35-27.54) | 59.4(47.54-72.39) | 206.50% | 26.53(20.83-38.29) | 41.72(33.89-50.09) | 1.39(1.11,1.67) |
| Viet Nam | Female | 119.29(94.58-148.22) | 236.88(178.87-304.74) | 98.57% | 49.71(39.48-61.66) | 43.85(33.38-55.72) | -0.58(-0.67,-0.50) |
| Afghanistan | Male | 17.41(8.16-31.87) | 27.53(14.77-47.25) | 58.13% | 44.73(21.32-80.88) | 34.49(19.14-56.84) | -0.95(-1.00,-0.90) |
| Armenia | Male | 8.05(6.94-9.41) | 9.26(7.53-11.18) | 15.03% | 58.65(50.96 -67.86) | 50.43(41.47-60.34) | -0.63(-0.98,-0.28) |
| Azerbaijan | Male | 7.11(5.71-8.9) | 17.75(12.6-24.14) | 149.65% | 29.31(23.62 -36.83) | 36.83(26.77-49.70) | 1.32(0.82,1.82) |
| Bahrain | Male | 0.77(0.62-0.94) | 2.44(1.70-3.32) | 216.88% | 61.30(48.51-76.19) | 31.27(22.81-41.25) | -2.82(-3.17,-2.48) |
| Bangladesh | Male | 689.11(448.69-956.45) | 899.42(607.55-1408.00) | 30.52% | 234.52(154.29 -323.89) | 127.11(86.41-197.85) | -2.19(-2.33,-2.03) |
| Bhutan | Male | 3.09(1.82-4.44) | 4.72(2.97-7.34) | 52.75% | 197.94(117.79 -283.56) | 148.14(95.37-224.46) | -1.06(-1.24,-0.90) |
| Brunei Darussalam | Male | 0.96(0.76-1.18) | 1.78(1.44-2.18) | 85.42% | 164.14(128.58 -201.57) | 110.91(91.45-134.25) | -1.10(-1.42,-0.78) |
| Cambodia | Male | 21.50(14.35-32.34) | 57.63(41.39-78.3) | 168.05% | 95.09(64.28 -142.83) | 102.73(73.36-137.18) | 0.26(0.16,0.37) |
| China | Male | 1385.95(1123.46-1664.77) | 4547.85(3602.61-5714.85) | 228.14% | 29.96(24.49 -35.71) | 45.75(36.55-56.94) | 2.42(2.02,2.81) |
| Cyprus | Male | 1.62(1.34-1.92) | 3.06(2.55-3.65) | 88.89% | 42.51(35.34-50.40) | 34.84(29.06-41.39) | -0.72(-0.83,-0.62) |
| Democratic People's Republic of Korea | Male | 40.76(26.8-59.64) | 74.47(51.42-108.07) | 82.70% | 51.30(35.06 -72.85) | 49.74(35.52-70.49) | 0.01(-0.05,0.07) |
| Georgia | Male | 22.64(18.57-28.04) | 27.71(22.85-33.25) | 22.39% | 82.71(68.67 -102.00) | 115.90(95.37-139.38) | 1.88(1.24,2.53) |
| India | Male | 5906.24(4851.81-7111.52) | 12654.1(9691.82-16055.69) | 114.25% | 208.51(168.72 -252.02) | 203.16(156.31-257.00) | -0.13(-0.22,-0.03) |
| Indonesia | Male | 315.05(254.13-379.20) | 731.87(522.11-1008.87) | 132.30% | 56.56(45.85 -67.24) | 64.03(46.64-87.82) | 0.5(0.45,0.55) |
| Iran(Islamic Republic of) | Male | 33.44(26.90-40.69) | 71.34(64.30-79.79) | 113.34% | 21.50(17.12-26.11) | 18.27(16.48-20.42) | -0.61(-0.68,-0.54) |
| Iraq | Male | 15.83(11.93-20.72) | 35.91(25.98-48.66) | 126.85% | 35.56(26.57-46.49) | 26.69(19.87-34.91) | -1.27(-1.38,-1.17) |
| Israel | Male | 5.50(5.00-6.02) | 14.39(12.78-16.03) | 161.64% | 25.28(22.91-27.77) | 28.51(25.37-31.81) | 0.08(-0.23,0.40) |
| Japan | Male | 298.98(292.24-305.11) | 480.10(445.04-503.02) | 60.58% | 38.79(37.85 -39.58) | 38.29(35.96-39.96) | -0.27(-0.61,0.08) |
| Jordan | Male | 3.73(2.88-4.72) | 12.77(9.46-16.95) | 242.36% | 43.41(33.84-54.13) | 31.10(23.21-40.66) | -1.30(-1.44,-1.17) |
| Kazakhstan | Male | 73.78(66.1-91.84) | 75.59(63.09-90.16) | 2.45% | 128.73(-159.62) | 93.55(78.48-110.34) | -1.53(-1.77,1.31) |
| Kuwait | Male | 1.61(1.39-1.84) | 3.14(2.42-4.02) | 95.03% | 30.40(26.17-34.69) | 17.12(13.33-21.82) | -1.58(-1.92,-1.24) |
| Kyrgyzstan | Male | 15.79(13.60-18.39) | 14.76(12.39-17.69) | -6.52% | 112.62(97.59 -130.92) | 63.11(52.99-75.33) | -1.62(-1.86,-1.37) |
| Lao People's Democratic Republic | Male | 12.04(7.12-19.09) | 18.2(12.54-24.16) | 51.16% | 105.42(62.58 -164.54) | 75.07(52.96-97.55) | -1.35(-1.49,-1.21) |
| Lebanon | Male | 6.72(4.57-8.92) | 10.80(7.94-14.71) | 60.71% | 54.14(37.00-70.85) | 45.59(33.50-62.58) | -0.14(-0.49,0.21) |
| Malaysia | Male | 44.35(35.98-54.89) | 103.17(75.88-137.00) | 132.63% | 85.66(69.79 -104.72) | 70.67(52.13-93.84) | -1.23(-1.66,-0.82) |
| Maldives | Male | 0.45(0.30-0.65) | 0.85(0.68-1.06) | 88.89% | 79.97(53.88 -114.26) | 45.46(35.99-56.84) | -2.58(-2.79,-2.37) |
| Mongolia | Male | 8.10(6.24-10.30) | 11.7(8.66-15.78) | 44.44% | 154.84(120.09 -195.34) | 92.46(69.85-122.19) | -2.79(-3.23,-2.35) |
| Myanmar | Male | 107.74(68.97-165.59) | 164.79(118.93-218.91) | 52.95% | 86.08(55.66 -132.14) | 73.54(53.63-96.53) | -0.58(-0.63,-0.53) |
| Nepal | Male | 97.83(60.02-142.50) | 160.4(117.42-209.70) | 63.96% | 169.80(105.29 -246.71) | 141.24(104.81-182.91) | -0.58(-0.97,-0.20) |
| Pakistan | Male | 1336.81(1118.55-1613.09) | 3375.03(2383.92-4648.57) | 152.47% | 386.40(324.32 -467.59) | 465.64(329.94-635.07) | 0.47(0.17,0.76) |
| Palestine | Male | 1.22(0.83-1.77) | 2.78(2.27-3.37) | 127.87% | 27.85(18.87-40.53) | 20.96(17.25-25.21) | -1.12(-1.28,-0.98) |
| Philippines | Male | 155.13(131.93-181.17) | 282.7(212.52-370.15) | 82.23% | 87.52(74.33 -104.33) | 67.53(51.22-87.73) | -1.30(-1.52,-1.09) |
| Qatar | Male | 0.35(0.25-0.47) | 2.15(1.45-3.04) | 514.29% | 32.70(23.55-43.14) | 21.25(15.23-28.74) | -1.54(-1.78,-1.30) |
| Republic of Korea | Male | 56.43(51.97-61.1) | 122.45(105.75-140.19) | 116.99% | 37.83(35.16 -40.93) | 29.61(25.74-33.78) | -1.50(-2.08,-0.93) |
| Saudi Arabia | Male | 12.30(8.39-16.51) | 41.17(28.91-57.31) | 234.72% | 28.67(19.86-37.70) | 25.67(18.94-34.35) | -0.52(-0.63,-0.41) |
| Singapore | Male | 7.32(6.70-7.93) | 10.41(9.12-11.97) | 42.21% | 63.49(58.08 -68.96) | 26.16(23.01-29.86) | -2.93(-3.13,-2.71) |
| Sri Lanka | Male | 70.91(61.69-81.57) | 173.25(118.09-237.81) | 144.32% | 123.16(107.70 -140.53) | 147.19(101.10-200.73) | 0.89(0.52,1.28) |
| Taiwan China | Male | 125.03(118.89-131.40) | 640.38(483.21-858.07) | 412.18% | 131.70(125.04-138.26) | 358.50(271.91-480.90) | 3.66(2.91,4.40) |
| Tajikistan | Male | 4.07(3.11-5.90) | 7.44(5.77-9.61) | 82.80% | 30.42(22.68 -46.57) | 28.13(22.30-35.69) | -0.33(-0.69,0.02) |
| Thailand | Male | 238.19(197.79-282.58) | 440.7(312.28-594.92) | 85.02% | 122.01(101.24-144.89) | 91.57(65.40-122.68) | -1.60(-1.85,-1.34) |
| Timor-Leste | Male | 0.95(0.56-1.41) | 2.52(1.43-3.62) | 165.26% | 50.89(30.71 -73.74) | 58.17(33.98-82.57) | 0.62(0.28,0.97) |
| Turkmenistan | Male | 9.61(8.79-10.54) | 18.3(14.28-23.23) | 90.43% | 99.04(90.94 -108.49) | 87.30(68.11-109.88) | -0.78(-1.05,-0.51) |
| United Arab Emirates | Male | 3.32(2.00-4.95) | 26.02(14.37-46.27) | 683.73% | 60.87(32.95-87.75) | 43.01(26.05-69.69) | -1.50(-1.71,-1.30) |
| Uzbekistan | Male | 28.94(24.36-37.16) | 94.55(77.79-113.42) | 226.71% | 48.57(40.64 -65.05) | 77.77(64.35-92.67) | 1.40(0.99,1.80) |
| Viet Nam | Male | 229.13(174.98-297.28) | 761.22(552.91-1000.51) | 232.22% | 122.28(93.82 -157.03) | 159.20(118.83-205.15) | 0.98(0.95,1.03) |
